# Targeting PLK1 potentiates the antitumor efficacy of EGFR-TKIs through inhibiting the JAK1/STAT3 pathway

**DOI:** 10.1038/s41419-025-08220-9

**Published:** 2026-01-15

**Authors:** Cheng Li, Shangxuan Shi, Long Li, Yafang Wang, Mingyue Yao, Chengcheng Yu, Chuwei Yu, Chengying Xie

**Affiliations:** 1 School of Life Science and Technology, ShanghaiTech University, Shanghai, China; 2 Shanghai Institute for Advanced Immunochemical Studies, ShanghaiTech University, Shanghai, China; 3 Lingang Laboratory, Shanghai, China

**Keywords:** Cancer therapeutic resistance, Tumour biomarkers

## Abstract

Despite the rapid development of epidermal growth factor receptor tyrosine kinase inhibitors (EGFR-TKIs) in recent decades, resistance remains a significant challenge in managing advanced non-small cell lung cancer (NSCLC). Elucidating the mechanisms underlying EGFR-TKI resistance and developing novel strategies are therefore crucial. In this study, we investigated the role of polo-like kinase 1 (PLK1) in EGFR-mutant NSCLC and evaluated the therapeutic potential of combining EGFR-TKIs with PLK1 inhibitors. We demonstrated that high PLK1 expression correlates with STAT3 signaling activation and decreased survival probability in EGFR-mutant NSCLC patients. Subsequent studies revealed that PLK1 inhibitors effectively reversed the activation of STAT3 induced by EGFR-TKIs. When used in combination with EGFR-TKIs, they promoted cell apoptosis, inhibited cell proliferation in vitro, and induced tumor regression in animal models. Mechanistically, our data demonstrated that PLK1 regulated STAT3 activity through protein-protein interactions and JAK1-mediated phosphorylation, while STAT3 reciprocally regulated PLK1 transcription, establishing a positive feedback loop between these signaling molecules. This PLK1/STAT3 loop was further reinforced by FGFR1 upregulation and directly linked to EGFR-TKI resistance. Targeting this axis with combinatorial inhibitors exerted synergistic anti-tumor effects, suppressing proliferation and migration in osimertinib-resistant models. In conclusion, concurrent inhibition of EGFR and FGFR1/STAT3/PLK1 signaling pathways provides a promising therapeutic strategy for NSCLC patients with EGFR mutations, enhancing efficacy and overcoming resistance.

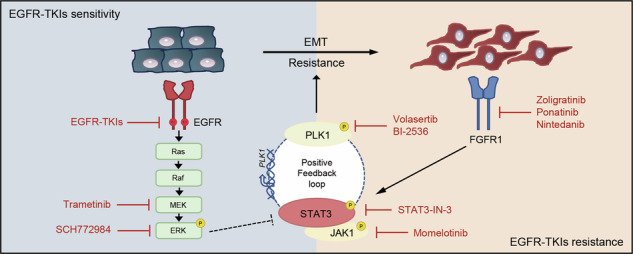

## Introduction

Non-small cell lung cancer (NSCLC) comprises adenocarcinoma, squamous cell carcinoma, and large cell carcinoma, collectively accounting for nearly 85% of lung cancer cases [1]. Despite advancements in treatment modalities—from conventional radiotherapy and chemotherapy to targeted therapy and immunotherapy, the survival rate for advanced NSCLC patients remains significantly low, which highlights the need for more effective treatment strategies [2, 3]. The epidermal growth factor receptor (EGFR) has emerged as a pivotal therapeutic target for NSCLC with EGFR mutations [4]. Approximately 19% of NSCLC patients carry various EGFR mutations [5]. Over the past few decades, EGFR tyrosine kinase inhibitors (EGFR-TKIs) have undergone rapid development, with three generations of EGFR-TKIs currently available for clinical use and several fourth-generation agents undergoing clinical trials [6–9].

The third-generation EGFR-TKIs, such as osimertinib, effectively overcome acquired EGFR T790M mutation-mediated resistance, yet treatment resistance inevitably develops within 18-19 months [10]. The C797S mutation in exon 20 of EGFR is one of the primary mechanisms among those secondary mutations that mediate acquired resistance to osimertinib treatment [11], while multiple off-target resistance pathways, including bypass signaling activation, downstream pathway alterations, and histomorphological alterations, also contribute to resistance [11, 12]. Signal transducer and activator of transcription 3 (STAT3), a critical transcription factor that operates downstream of tyrosine kinase receptors, plays an essential role in mediating growth factor- and cytokine-activated signaling pathways [13]. Emerging evidence indicates that activation of STAT3 is one of the contributors to EGFR-TKI resistance, and targeted inhibition of STAT3 signaling has shown potential to circumvent therapeutic resistance both in lung cancer and other malignancies [14, 15]. However, the complexity of STAT3-mediated resistance mechanisms, including its dynamic interplay with other signaling pathways, necessitates further comprehensive investigation to fully elucidate its role in EGFR-TKIs therapeutic resistance. The limitations of EGFR mutation-targeted monotherapy in NSCLC underscore the need for combined therapeutic strategies, specifically combinatorial regimens that integrate pathway modulation and phenotypic targeting [16].

Polo-like kinase 1 (PLK1), a serine/threonine kinase, acts as a central regulator of mitotic progression, orchestrating critical processes from initiation to completion of mitotic and playing a pivotal role in cell cycle regulation [17]. In addition, accumulating evidence has substantiated PLK1’s involvement in diverse cellular processes, including DNA damage response, autophagy regulation, and apoptosis modulation [18–20]. Reflecting its fundamental biological roles, PLK1 exhibits elevated mRNA and protein expression in proliferating tissues and frequently exhibits overexpression across multiple malignancies, which is strongly associated with poor outcomes [21]. The oncogenic significance of PLK1 is further underscored by its association with chemotherapy resistance, and its inhibition has been shown to enhance the sensitivity of cancer cells to both chemotherapy and radiotherapy [22, 23]. In the context of NSCLC, extensive preclinical and clinical studies have demonstrated PLK1 overexpression, establishing its potential as a promising diagnostic marker and therapeutic target [24–28]. The therapeutic potential of PLK1 inhibition has driven the development of two distinct classes of small-molecule inhibitors targeting PLK1, encompassing ATP-competitive inhibitors and non-ATP competitive inhibitors that specifically target the polo-box domain (PBD) [29]. Among these, ATP-competitive inhibitors such as BI-2536, volasertib, and plogosertib, have demonstrated efficacy in preclinical and clinical studies for advanced solid tumors, including NSCLC [29–31]. Although non-ATP competitive inhibitors targeting the PBD offer theoretical advantages in selectivity and reduced toxicity due to their unique binding mechanism [32], their development has been constrained by inadequate efficacy, as exemplified by rigosertib, a compound with favorable safety profiles but limited clinical activity when used as alone in clinical trials for NSCLC [33].

Despite promising preclinical and early clinical trial results, the clinical application of PLK1 inhibitors as single agents has been limited by toxicity concerns and narrow therapeutic windows [29]. Further investigations are imperative to gain a more comprehensive understanding of the regulatory mechanisms governing PLK1, its crosstalk with oncogenic signaling pathways, and its multifaceted roles in non-cell cycle processes. Developing PLK1 inhibitors with improved safety profiles and enhanced therapeutic efficacy remains an urgent clinical need, while rational combination strategies targeting multiple pathways hold significant therapeutic promise [34]. Notably, the third-generation PLK1 inhibitor onvansertib has entered the Phase II clinical trials and shown significant efficacy as a second-line treatment for KRAS-mutant metastatic colorectal cancer when combined with standard care (FOLFIRI/bevacizumab) [35].

In this study, we aim to investigate the therapeutic potential of PLK1 inhibition in EGFR-mutant NSCLC, aiming to elucidate the mechanisms underlying the synergistic antitumor effects achieved through co-targeting the EGFR/ERK and PLK1/STAT3 signaling pathways, as well as the capacity of these synergistic effects to overcome acquired resistance to EGFR-TKIs.

## Materials and methods

### Cell lines and cell culture

PC9 were originally obtained from the European Collection of Authenticated Cell Cultures (ECACC), and NCI-H1975 from the American Type Culture Collection (ATCC). Both cell lines were cultured in DMEM (C11995500CP, Gibco, Thermo Fisher Scientific, Waltham, USA) medium with 10% fetal bovine serum (FBS, FB-1058/500, Biosera, Epsom, UK). Ba/F3 cells transfected with plasmids expressing various EGFR mutations were grown in RPMI-1640 (C22400500CP, Gibco) medium with 10% FBS; otherwise supplemented with 10% interleukin-3 (IL-3) in the case of the Ba/F3 cell line. The cell lines were routinely confirmed to be mycoplasma negative and authenticated using the short tandem repeat method based on ECACC or ATCC profiles. The osimertinib-resistant PC9/OR cells were constructed by gradually increasing the concentrations of osimertinib in PC9 parental cells, and monoclonal cell lines, such as PC9/OR3, were derived from the PC9/OR cells.

### Apoptosis assay

Annexin V-FITC/propidium iodide (PI) double-staining apoptosis detection kit (C1062L, Beyotime, Shanghai, China) was used to quantify the percentage of apoptotic cells. Cells were collected and dyed according to the manufacturer’s instructions, and then assayed using CytoFLEX (Beckman, California, USA). The proportion of apoptotic cells was analyzed using FlowJo software.

### Co-immunoprecipitation (Co-IP) and mass spectrometry (MS)

Cell lysates were incubated with normal rabbit IgG (NI01-100UGCN, Merck/Millipore, Darmstadt, Germany), anti-PLK1 (#4513, Cell Signaling Technology, Danvers, USA), or anti-STAT3 (#12640, Cell Signaling Technology) antibodies overnight at 4 °C. This was followed by incubation with protein A/G agarose beads (#9863, #37478, Cell Signaling Technology) for 4 h at 4 °C. Immunoprecipitated proteins were separated from the supernatants by centrifugation and analyzed by Western blot.

The Co-IP samples were concentrated using SDS-PAGE, followed by cutting the selected gels, dehydration, vacuum drying, and incubation with trypsin at 37 °C and 500 rpm overnight enzyme digestion. The proteins post enzyme digestion were vacuum-dried, desalted, and then sent to the omics analysis platform of the College of Life Sciences, Shanghaitech University for liquid chromatoc-tandem mass spectrometry (LC/MS-MS) analysis.

### Nuclear and cytoplasmic protein extraction assay

NCI-H1975 cells were treated with PLK1 inhibitors in 10 cm-dish for 24 h. Following treatment, the cells were lysed on ice and then centrifuged at 1500 × *g*, for 10 min at 4 °C to obtain the supernatant, which contained cytoplasmic proteins. The remaining precipitate was disrupted using 0.2 M HCl (containing 10% glycerol) and then centrifuged at 12,000 rpm for 15 min at 4 °C, yielding a supernatant containing nuclear proteins. Changes in the expression of the corresponding proteins were confirmed by Western blot analysis.

### RNA sequencing (RNA-seq)

Total RNA was extracted using TRIzol reagent (15596018, Thermo/Life/invitrogen). RNA-seq and data processing were provided by NEO BIO (Shanghai, China) or Majorbio Biotech (Shanghai, China). The Illumina HiSeq sequencing platform was used for the high-throughput sequencing of the samples. Differentially expression analysis was performed using DESeq2. Differentially expressed genes were analyzed by Gene Ontology (GO) function and Kyoto Encyclopedia of Genes and Genomes (KEGG) pathway enrichment using DAVID (https://david.ncifcrf.gov/tools.jsp).

### Animal study

Female nude mice (BALB/c nude, 5–6 weeks old) were purchased from Beijing Vitonglihua Experimental Animal Technology Co., LTD (Beijing, China). All in vivo experiments were performed according to the Institutional Ethical Guidelines on Animal Care and were approved by the Institute of Animal Care and Use Committee at Lingang Laboratory. Single-cell suspensions were implanted subcutaneously into the right flank of mice. The mice were randomly allocated to the control, monotherapy, and combined treatment groups (five mice per group). Volasertib was administered orally on a weekly basis and osimertinib was administered orally daily. Tumors were measured every three days by microcalipers and individual tumor volumes were calculated as follows: ½ × length × width^2^.

### Additional materials and methods

Additional methods are described in Supplementary Methods.

## Results

### PLK1 inhibitors exhibit broad-spectrum antiproliferative activity and clinical potential for EGFR-mutant NSCLC

**Fig. 1:**
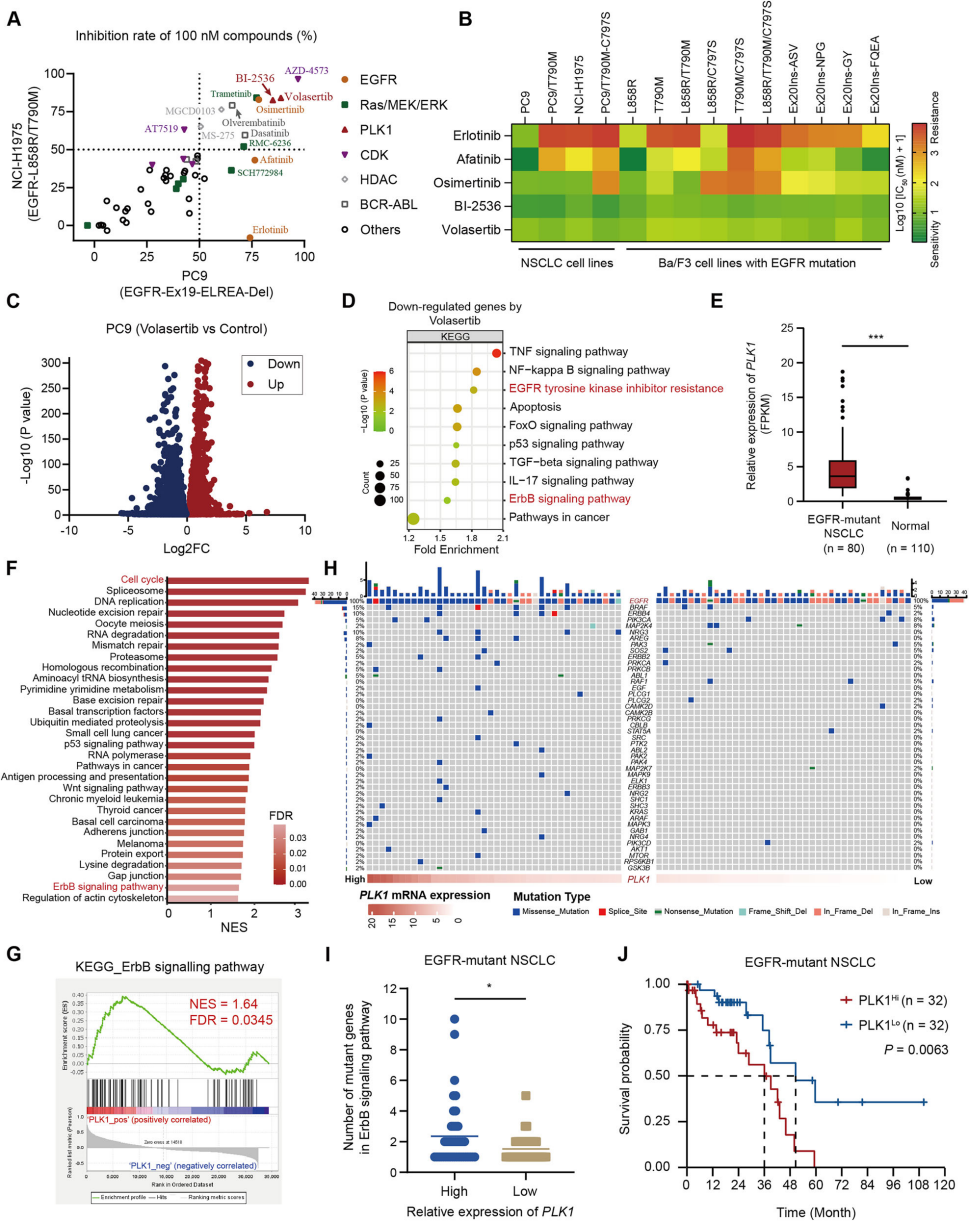
PLK1 inhibitors exhibit broad-spectrum antiproliferative activity and clinical potential for EGFR-mutant NSCLC. **A** Screening of compound library was performed to assess the anti-proliferation activity in PC9 and NCI-H1975 cells at a concentration of 100 nM for 5 days. **B** IC_50_ values of PLK1 inhibitors and EGFR-TKIs were measured in EGFR-mutant NSCLC and Ba/F3 cell lines with different EGFR mutations following treatment for 5 days. **C** The volcano plot analysis of the differential gene expression in PC9 cells following treatment with 40 nM volasertib for 24 h, as compared to control (|Log2FC|> 0 and *P* < 0.05). **D** KEGG enrichment analysis of downregulated genes by volasertib (Log2FC < 0 and *P* < 0.05). **E** Relative PLK1 mRNA expression in tumor tissues was compared to adjacent normal tissues from EGFR-mutant NSCLC patients, as assessed using the Wilcoxon test (***P < 0.001). **F** Gene expression data of EGFR-mutant NSCLC patients (n = 80) from the TCGA database were utilized for conducting gene set enrichment analysis (GSEA) on *PLK1* gene (NES > 1, FDR < 0.05). **G** GSEA analysis of the correlation between the *PLK1* gene and ErbB signaling pathway in EGFR-mutant NSCLC patients. **H** A mutation information matrix of genes associated with the ErbB signaling pathway was constructed for NSCLC patients harboring EGFR mutations and stratified by high or low *PLK1* mRNA expression (n = 80). **I** Cumulative mutations of the ErbB signaling pathway genes were compared between high and low PLK1 expression groups, as assessed using a two-tailed unpaired Student’s *t* test (*P < 0.05). **J** Kaplan-Meier survival curves of EGFR-mutant NSCLC patients stratified by PLK1 expression after PSM to exclude the effects of stage.

Given the limited efficacy and the development of resistance to EGFR-TKIs, we performed compound library screening on PC9 (EGFR exon 19 ELREA-Del) and NCI-H1975 (EGFR L858R/T790M) cell lines to identify potential targets in EGFR-mutant NSCLC. This screening utilized a collection of molecular targeted agents that are either approved or in clinical development. Our results identified several classes of targeted inhibitors with prominent antiproliferative effects, including targeting EGFR, RAS/MEK/ERK, PLK1, CDKs, HDAC, and BCR-ABL, as potential therapeutic agents, based on their prominent antiproliferative effects (>50% at 100 nM) (Fig. 1A and Supplementary Table S1). Notably, both PLK1 inhibitors (volasertib and BI-2536) showed marked antiproliferative activity in EGFR-mutated NSCLC cell lines. BI-2536 has underwent clinical trials for NSCLC [31], although its mechanism of action in the context of EGFR mutations remains unclear. Based on these findings, PLK1 was identified as a key therapeutic target for further investigation in EGFR-mutant NSCLC. To assess the inhibitory effects of PLK1 inhibitors and EGFR-TKIs, we established cell models including PC9/T790M, PC9/T790M-C797S, and Ba/F3 cell lines harboring diverse EGFR mutations. The EGFR-TKI sensitivity of these cell lines aligned with established patterns, with varied responses across different mutations [36]. However, PLK1 inhibitors exhibited broad-spectrum antiproliferative activity in various EGFR-mutant NSCLC and Ba/F3-transformed cell lines, including those with secondary mutations, with average IC_50_ values ranging from 5 to 10 nM (Fig. 1B and Supplementary Fig. 1A).

To gain deeper insight into the antitumor activity of PLK1 inhibitors in EGFR-mutant NSCLC cells, we performed RNA-seq to profile differentially expressed genes (DEGs) in PC9 cells treated with the PLK1 inhibitor volasertib. Comparative analysis revealed that volasertib significantly downregulated 4232 genes and upregulated 5474 genes (Fig. 1C). Additionally, KEGG enrichment analysis showed that downregulated genes were predominantly associated with PLK1-related signaling pathways, including apoptosis, NF-κB signaling, TGF-β signaling, EGFR-TKI resistance and the ErbB signaling pathway (Fig. 1D). Volasertib-induced apoptosis in PC9 cells was validated by annexin-V/PI double staining (Supplementary Fig. 1B). Collectively, these findings indicate a correlation between PLK1 and EGFR signaling, potentially providing a rationale for combinational drug strategies.

To further determine the role of PLK1 in EGFR-mutant NSCLC, we conducted a comprehensive analysis using clinical databases. Immunohistochemical data from the HPA database, encompassing both lung adenocarcinoma (LUAD) and lung squamous cell carcinoma (LUSC), revealed significantly higher PLK1 expression in NSCLC patient tumor tissues compared to normal tissues (Supplementary Fig. 1C). Additionally, we utilized TCGA database to compile transcriptome data, gene mutation information, and clinical survival data from EGFR-mutant NSCLC patients. As shown in Fig. 1E, *PLK1* mRNA expression was markedly elevated in the tumor tissues versus adjacent normal tissues from EGFR-mutant NSCLC patients. Gene set enrichment analysis (GSEA) of transcriptomic data from 80 EGFR-mutated NSCLC patients demonstrated that higher *PLK1* mRNA expression positively correlated with activation of both canonical cell cycle-related signaling pathways and the ErbB signaling pathway (Figs. 1F, G). We then stratified EGFR-mutant NSCLC patients by *PLK1* mRNA expression (sorted from high to low), defining those with expression above the median as the PLK1-high group and those below as the PLK1-low group. Notably, the number of patients with late-stage (III and IV) NSCLC in the PLK1-high group was more than twice that in the PLK1-low group (Supplementary Fig. 1D). Furthermore, we mapped ErbB signaling pathway genes mutations in EGFR-mutant NSCLC patients (Fig. 1H). Clearly, higher *PLK1* mRNA expression corresponded to a greater cumulative number of ErbB signaling-related mutated genes in these patients (Fig. 1I). Additionally, after using the propensity score matching (PSM) method to eliminate the confounding effect of tumor stage on survival outcomes, Kaplan-Meier survival curves were plotted in EGFR-mutant NSCLC patients. The results indicated that the survival probability in the PLK1-high group decreased more rapidly, and the prognosis was poorer. The survival difference between the two groups was statistically significant (*P* = 0.0063), suggesting that the PLK1 expression level might be a potential predictor of survival prognosis in EGFR-mutant NSCLC patients (Fig. 1J). Taken together, targeting PLK1 represents a potential approach for the treatment of EGFR-mutant NSCLC.

### Combining PLK1 inhibitors with EGFR-TKIs exhibits synergistic efficacy in EGFR-mutant NSCLC both in vitro and in vivo

**Fig. 2:**
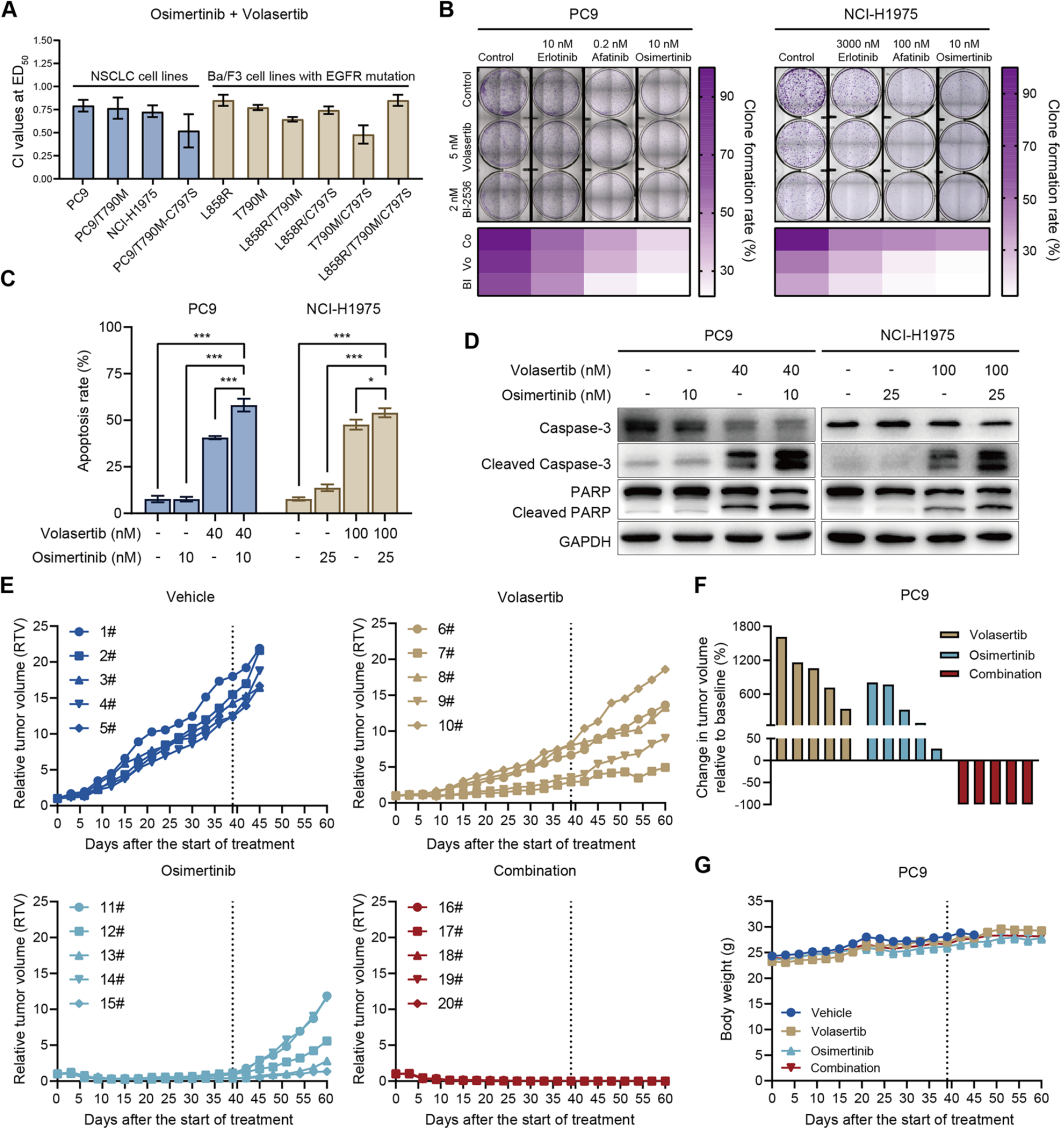
Combining PLK1 inhibitors with EGFR-TKIs exhibits synergistic efficacy in EGFR-mutant NSCLC both in vitro and in vivo. **A** The combined effects of volasertib and osimertinib were evaluated using Calcusyn software to calculate the combination index (CI) values in various cell lines with EGFR mutations. **B** Clonogenic assay of PC9 and NCI-H1975 cells following PLK1 inhibitors combined with EGFR-TKIs treatment for 7 days. **C** PC9 and NCI-H1975 cells were treated with volasertib, osimertinib or their combination for 48 h, and cell apoptosis was assessed using flow cytometry (*P < 0.05, **P < 0.01, ***P < 0.001). **D** Western blot analysis of apoptosis-related proteins in PC9 and NCI-H1975 cells following treatment with volasertib combined with osimertinib for 48 h. **E** Relative tumor volume (RTV) was measured for PC9 xenografts treated with vehicle, volasertib (20 mg/kg), osimertinib (2.5 mg/kg), or their combination. Dosing for each group was ceased on day 39, and continuous observation was maintained until day 60 (n = 5 mice per group). **F** Tumor volume changes of individual mice from the PC9 xenograft model at day 60 were shown as a waterfall plot. **G** The body weights of mice were recorded every three days throughout the experiment.

Given that PLK1 inhibitors exhibit strong antiproliferative activity in NSCLC cells and have clinical relevance in EGFR-mutated patients, we further evaluated the efficacy of their combination with EGFR-TKIs to enhance therapeutic effects and reduce potential toxic side effects. First, we examined the effects of combining volasertib with osimertinib on cell proliferation. Drug synergy was quantified using Calcusyn software to calculate the CI value. The combination of volasertib and osimertinib demonstrated significant synergistic inhibition of proliferation in various EGFR-mutant cell lines, including those with secondary mutations (CI < 1, Fig. 2A). Clonogenic assays further confirmed the synergistic inhibition of cell proliferation upon combination of the PLK1 inhibitors volasertib or BI-2536 with EGFR-TKIs (Fig. 2B). Moreover, co-treatment significantly increased apoptosis in both NCI-H1975 and PC9 cells compared to either agent alone (Fig. 2C). This was further confirmed by the elevated levels of cleaved caspases 3 and poly-(ADP-ribose) polymerase (PARP) following co-treatment (Fig. 2D).

Given the observation of the significant in vitro synergism of volasertib combined with osimertinib, we further assessed the in vivo antitumor potential of this combination using a PC9 xenograft model. As shown in Fig. 2E, all mice in the combination group achieved tumor regression by day 39, at which point drug administration was discontinued for all groups, and continuous observation was maintained. During experiment, osimertinib monotherapy showed substantial antitumor efficacy but failed to induce tumor regression; rapid tumor recurrence was observed after drug withdrawal. In contrast, the combination group maintained sustained tumor regression with no recurrence in all mice through day 60, which was distinct from the tumor regrowth seen in both monotherapy groups (Fig. 2E, F). Importantly, all treatment regimens were well-tolerated, as evidenced by the absence of significant body weight changes in mice across groups (Fig. 2G). Taken together, these findings highlight the potential of targeting PLK1 as a synergistic strategy with EGFR-TKIs, underscoring the therapeutic value of combining PLK1 inhibitors with EGFR-TKIs for treating NSCLC patients with various EGFR mutations, including those harboring secondary mutations.

### PLK1 inhibitors reverse the activation of STAT3 induced by EGFR/MEK/ERK inhibition

**Fig. 3:**
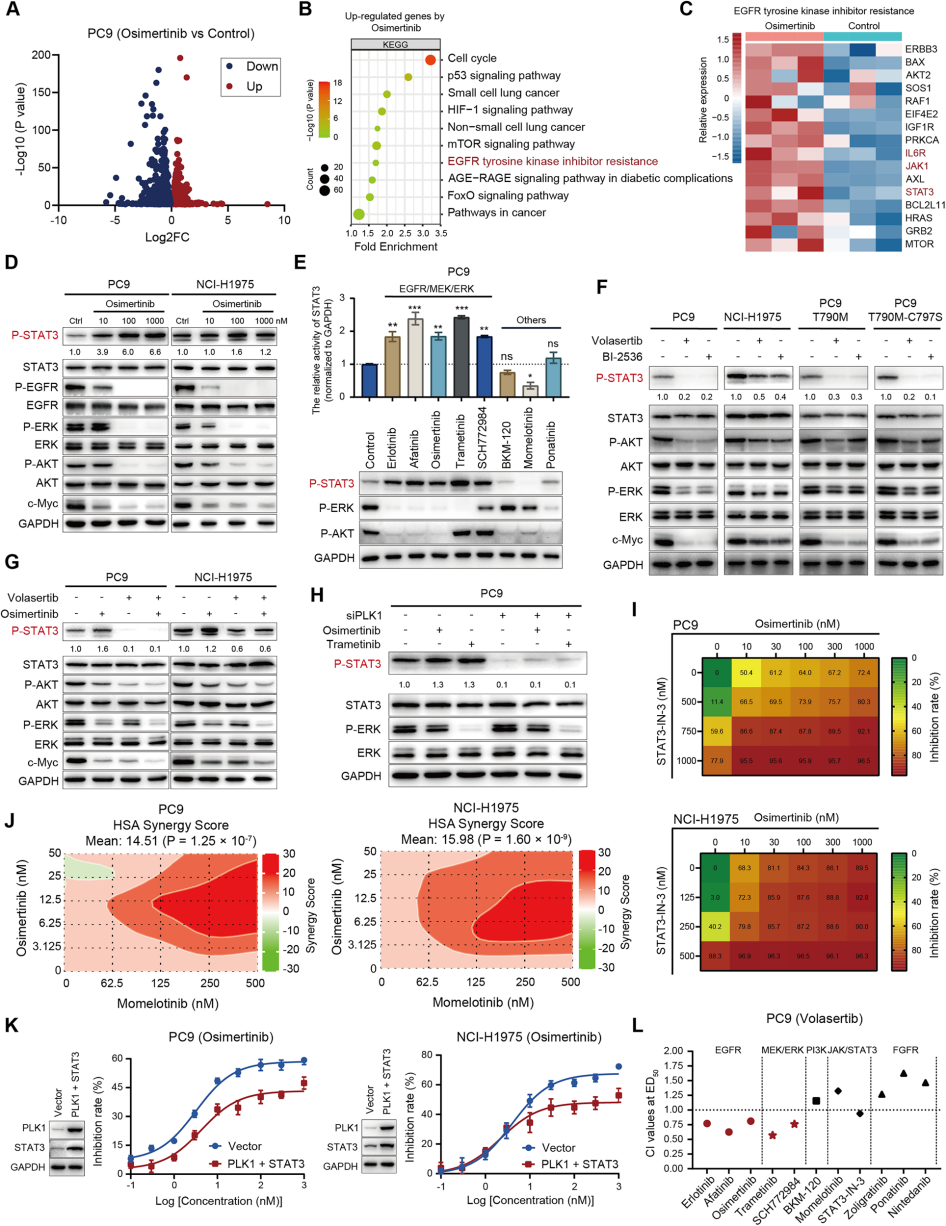
PLK1 inhibitors reverse the activation of STAT3 induced by EGFR/MEK/ERK inhibition. **A** The volcano plot analysis of the differential gene expression in PC9 cells following treatment with 10 nM osimertinib for 24 h, as compared to control (|Log2FC|> 0 and *P* < 0.05). **B** KEGG enrichment analysis of upregulated genes (Log2FC > 0 and *P* < 0.05) by osimertinib. **C** Heatmap analysis of STAT3 and associated genes within the EGFR tyrosine kinase inhibitor resistance signaling pathway. **D** Western blot analysis of EGFR signaling pathways in PC9 and NCI-H1975 cells following treatment with osimertinib for 24 h. **E** Western blot analysis of EGFR downstream effectors in PC9 cells following treatment with different compounds (1000 nM) for 24 h. The relative activity of STAT3 was quantitatively analyzed using Image J software (ns: no significance, *P < 0.05, **P < 0.01, ***P < 0.001). **F** Western blot analysis of EGFR downstream effectors in EGFR-mutant NSCLC cell lines following treatment with PLK1 inhibitors (100 nM) for 24 h. **G** Western blot analysis of EGFR signaling pathways in PC9 and NCI-H1975 cells following volasertib (40 nM) combined with osimertinib (10 nM) for 24 h. **H** Western blot analysis of STAT3 phosphorylation in PC9 cells following PLK1 knockdown combined with osimertinib (10 nM) or trametinib (10 nM) treatment for 24 h. **I** Cell proliferation assays of PC9 and NCI-H1975 cells following treatment with STAT3-IN3 and osimertinib, alone or in combination for 5 days. **J** The HSA model synergy analysis of JAK1/2 inhibitor momelotinib combined with osimertinib for 5 days in PC9 and NCI-H1975 cells. **K** Cell proliferation analysis was performed to assess the sensitivity of PC9 and NCI-H1975 cells to osimertinib after treatment with osimertinib for 3 days following exogenous co-overexpression of PLK1 (pCDNA3-PLK1) and STAT3 (pCDNA3-STAT3). **L** The CI values of volasertib with indicated compounds in PC9 cells treated for 5 days were calculated using Calcusyn software.

To explore the synergistic mechanism underlying the drug combination, we conducted RNA-seq analysis, which revealed 2077 genes upregulated in PC9 cells following osimertinib treatment (Fig. 3A). KEGG enrichment analysis of these upregulated genes further demonstrated significant enrichment in the EGFR-TKI resistance pathway (Fig. 3B). Consistent with previous studies showing that activation of STAT3 promotes cell survival and limits drug efficacy [15], heatmap analysis revealed coordinated upregulation of IL6R, JAK1, and STAT3 in osimertinib-treated PC9 cells (Fig. 3C), indicating that the JAK1/STAT3 signaling pathway is a potential mediator of EGFR-TKI resistance. Western blot analysis confirmed that the EGFR-TKIs osimertinib and afatinib inhibited EGFR, AKT, and ERK phosphorylation, while inducing STAT3 activation in EGFR-mutant NSCLC cell lines (Fig. 3D and Supplementary Fig. 2A). Notably, MEK/ERK inhibitors similarly induced STAT3 activation, whereas the PI3K inhibitor BKM-120, JAK1/2 inhibitor momelotinib, and FGFR1/Src/ABL inhibitor ponatinib did not elicit this response (Fig. 3E and Supplementary Fig. 2B).

While PLK1 inhibition is known to reduce EGFR protein levels and slightly decrease AKT and ERK phosphorylation, the mechanism underlying its EGFR-independent inhibition of STAT3 phosphorylation remains unclear [37]. Consistent with this, our results showed that the PLK1 inhibitors volasertib and BI-2536 effectively suppressed STAT3 phosphorylation, alongside AKT and ERK phosphorylation, and reduced c-Myc expression across all EGFR-mutant NSCLC cell lines, including those with acquired resistance mutations (Fig. 3F). Furthermore, volasertib reversed osimertinib-induced STAT3 activation, and the combination synergistically inhibited AKT and ERK phosphorylation as well as c-Myc expression in both PC9 and NCI-H1975 cells (Fig. 3G). Similar to pharmacological inhibition, PLK1 knockdown effectively reversed osimertinib- and trametinib-induced STAT3 activation (Fig. 3H).

To further investigate how aberrant JAK/STAT3 signaling modulates the sensitivity of EGFR-mutant NSCLC cells to EGFR-TKIs, we evaluated pharmacological and genetic interventions. STAT3-IN-3, a selective STAT3 inhibitor [38], markedly enhanced the sensitivity of EGFR-mutant NSCLC cell lines to osimertinib (Fig. 3I). Additionally, combining the JAK1/2 inhibitor momelotinib with osimertinib exhibited synergistic antiproliferative effects in PC9 and NCI-H1975 cells, as evidenced by an HSA synergy score exceeding 10 (Fig. 3J). Conversely, concurrent overexpression of PLK1 and STAT3 significantly reduced both cell lines’ sensitivity to osimertinib (Fig. 3K). These results indicate that PLK1 inhibitors improve the therapeutic efficacy by functionally reversing EGFR-TKI-induced STAT3 activation. This mechanistic relationship is further supported by the selective synergy between volasertib and agents triggering STAT3 activation (e.g., MEK/ERK inhibitors and EGFR-TKIs), but not with mechanistically distinct inhibitors (Fig. 3L). Collectively, these results underscore the critical role of PLK1 inhibition in counteracting STAT3 activation, thereby enhancing the antitumor effects of both EGFR-TKIs and MEK/ERK inhibitors.

### PLK1 contributes to the JAK1-mediated phosphorylation of STAT3

**Fig. 4:**
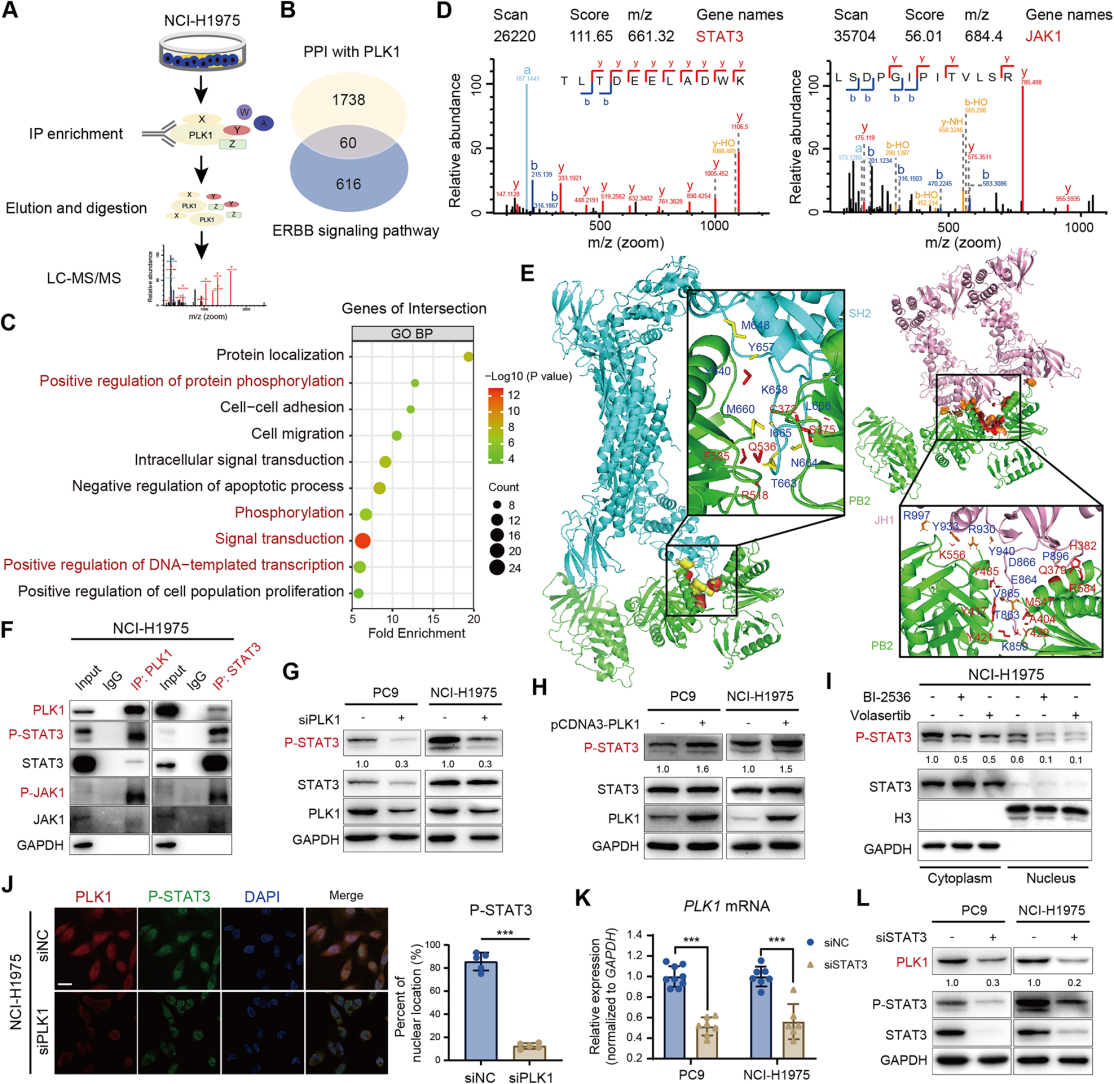
PLK1 contributes to the JAK1-mediated phosphorylation of STAT3. **A** A schematic workflow of label-free quantitative proteomics utilizing PLK1-immunoprecipitates derived from NCI-H1975 cells analyzed by LC-MS/MS. **B** Venny diagram analysis of PLK1-interacting proteins intersected with ErbB signaling pathways, including the MAPK, PI3K/AKT, and JAK/STAT signaling pathways. **C** GO/BP enrichment analysis of 60 overlapping proteins (*P* < 0.05, Top10). **D** Peak maps analysis of JAK1 and STAT3 peptides obtained from LC-MS/MS. **E** Structural modeling using ClusPro v2.0 predicted interaction between PLK1 (green), STAT3 (blue), and JAK1 (pink), with color-coded indicating potential binding sites (red: located in PLK1, orange: located in STAT3, yellow: located in JAK1). **F** NCI-H1975 cell lysates underwent immunoprecipitation using anti-PLK1 or anti-STAT3 antibodies, followed by Western blot analysis with the indicated antibodies, using normal rabbit IgG as a control. **G** Western blot analysis of STAT3 phosphorylation in PC9 and NCI-H1975 cells following PLK1 knockdown using siRNA for 48 h. **H** Western blot analysis of STAT3 phosphorylation in PC9 and NCI-H1975 cells following the exogenous overexpression of PLK1 (pCDNA3-PLK1) for 48 h. **I** Nuclear and cytoplasmic protein extraction assay of P-STAT3 expression in NCI-H1975 cells following treatment with PLK1 inhibitors (100 nM) for 24 h. Histone H3 was used as a nuclear loading control, whereas GAPDH served as a cytoplasmic loading control. (**J**) Immunofluorescence analysis of PLK1 and P-STAT3 in NCI-H1975 cells following PLK1 knockdown using siRNA for 48 h, with siNC used as the negative control. Scale bar, 20 μm. (**K**) RT-qPCR and (**L**) Western blot analysis of *PLK1* mRNA and protein expression in PC9 and NCI-H1975 cells following STAT3 knockdown using siRNA for 48 h (***P < 0.001).

Analysis of the TCGA database further revealed that co-expression of PLK1 and STAT3 significantly worsened survival prognosis in EGFR-mutant NSCLC patients but had no discernible impact in those with non-EGFR mutations (Supplementary Fig. 3A), indicating the critical role of PLK1-STAT3 interplay in EGFR-mutant NSCLC. To investigate the molecular mechanisms underlying this relationship, we performed immunoprecipitation coupled with LC-MS/MS-based proteomics in NCI-H1975 cells, identifying 1798 PLK1-interacting proteins (Fig. 4A). Given the positive correlation between PLK1 expression and the ErbB signaling pathway, we intersected the identified proteins with those involved in the ErbB pathway (encompassing the PI3K/AKT, MAPK, and JAK/STAT signaling pathways), yielding 60 common proteins (Fig. 4B and Supplementary Table S2).

GO/BP enrichment analysis demonstrated that the primary cellular biological functions influenced by these common proteins include protein phosphorylation, signal transduction, and transcription (Fig. 4C). Moreover, GO/MF enrichment analysis revealed that the molecular functions of these PLK1-interacting common proteins are primarily associated with various forms of protein binding (Supplementary Fig. 3B). A chord diagram illustrated extensive involvement of JAK1/STAT3 in these biological functions (Supplementary Fig. 3C). Specifically, LC-MS/MS identified six STAT3 peptides, five STAT1 peptides, and two JAK1 peptides (Fig. 4D and Supplementary Table S2). Given that JAK1 is a direct upstream regulator of STAT3 phosphorylation [39], we predicted potential interactions between PLK1 and JAK1/STAT3 using the ClusPro server and visualized these interactions with PyMOL. The results indicated that the PBD domain (PB2) of PLK1 interacts with the kinase domain (JH1) of JAK1 and the SH2 domain of STAT3, respectively. Amino acids on PLK1 within 4 Å of STAT3 and JAK1 are highlighted in red, suggesting these residues may mediate PLK1’s interaction with STAT3 and JAK1 (Fig. 4E). These PLK1-JAK1/STAT3 interactions were experimentally confirmed via immunoprecipitation using anti-PLK1 and anti-STAT3 antibodies (Fig. 4F).

Further studies using genetic manipulation of PLK1 expression revealed distinct regulatory mechanisms. While previous literature indicated that PLK1 modulates STAT3 transcription via β-catenin in esophageal cancer cell lines [40], our results showed that siRNA-mediated PLK1 knockdown only moderately reduced STAT3 transcription (Supplementary Fig. 3D). Western blot analysis revealed only a minor effect on basal STAT3 expression in PC9 cells and virtually no impact in NCI-H1975 cells (Fig. 4G). Importantly, PLK1 knockdown significantly decreased STAT3 phosphorylation (Fig. 4G), whereas PLK1 overexpression via the pCDNA3-PLK1 plasmid increased STAT3 phosphorylation in both PC9 and NCI-H1975 cells (Fig. 4H). Next, nuclear-cytoplasmic protein fractionation confirmed that PLK1 inhibitors markedly suppressed nuclear P-STAT3 abundance (Fig. 4I). Similarly, immunofluorescence analysis showed that PLK1 knockdown substantially reduced STAT3 phosphorylation and profoundly disrupted its nuclear localization (Fig. 4J). Collectively, these findings indicate that in EGFR-mutant NSCLC cells, PLK1 predominantly modulates STAT3 through a phosphorylation-dependent mechanism rather than by regulating basal expression levels.

Interestingly, siRNA-mediated STAT3 downregulation in PC9 and NCI-H1975 cells also significantly reduced both PLK1 transcription and protein expression (Fig. 4K, L), suggesting a reciprocal regulation between STAT3 and PLK1. Collectively, these findings demonstrate that PLK1 facilitates JAK1-mediated STAT3 phosphorylation, and its inhibition disrupts the STAT3-PLK1 regulatory axis, thereby enhancing EGFR-TKI-induced cell death in EGFR-mutant NSCLC.

### Abnormal FGFR1/STAT3/PLK1 axis mediates resistance to EGFR-TKIs in EGFR-mutant NSCLC

**Fig. 5:**
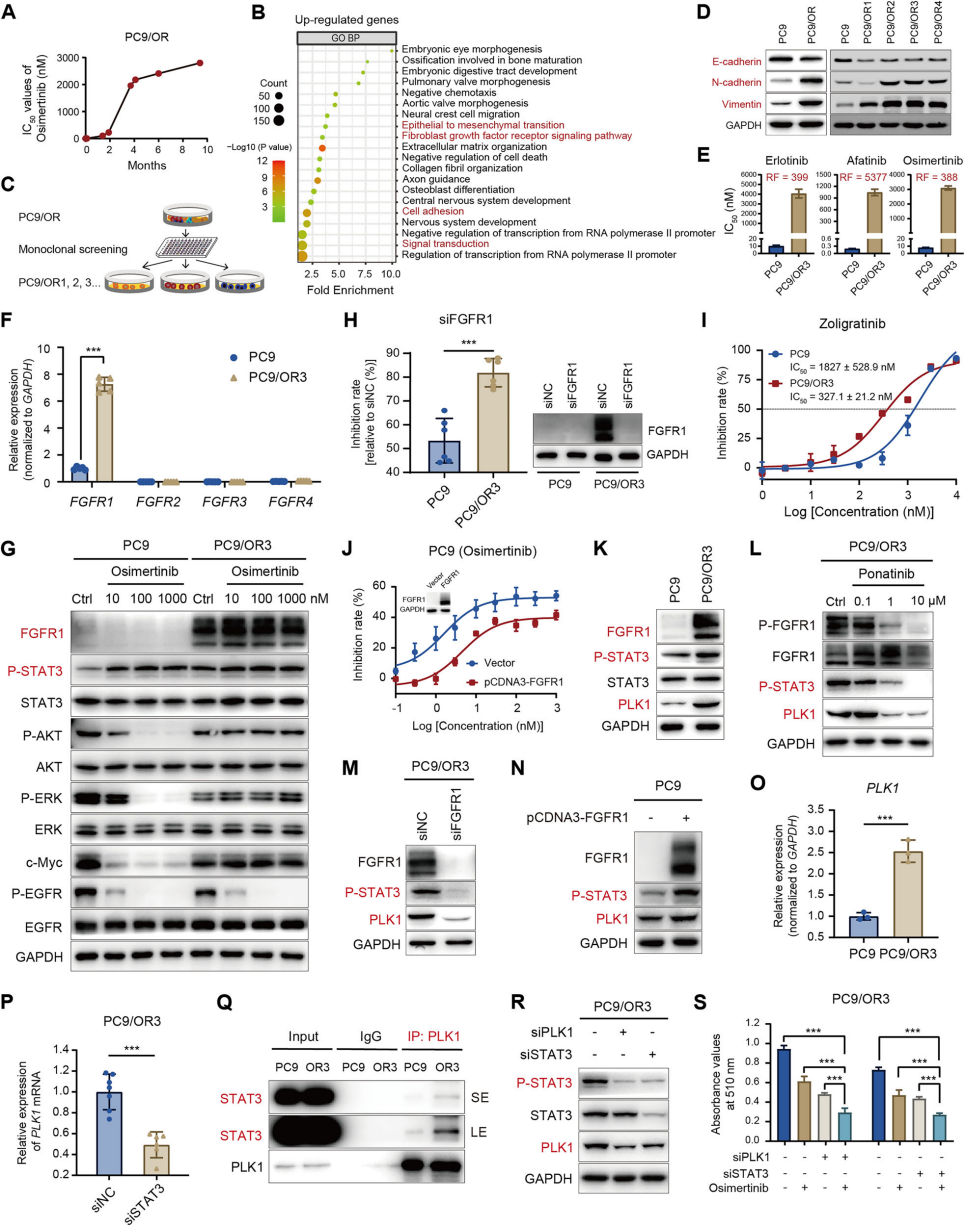
Abnormal FGFR1/STAT3/PLK1 axis mediates resistance to EGFR-TKIs in EGFR-mutant NSCLC. **A** The development timeline correlates with the progression of osimertinib resistance in PC9/OR cells with the IC_50_ values indicated. **B** GO/BP enrichment analysis of upregulated genes in PC9/OR cells compared to PC9 cells. (*P* < 0.05, Top20). **C** Schematic workflow for the screening of monoclonal resistant cell lines derived from PC9/OR. **D** Western blot analysis of EMT-related marker proteins (E-cadherin, N-cadherin, and Vimentin) in parental PC9 and acquired resistant cells. **E** IC_50_ values of EGFR-TKIs in PC9 and PC9/OR3 cells. The resistance factor (RF) was calculated as the ratio of IC_50_ values (resistant line/parental line). **F** RT-qPCR analysis of the relative expression levels of *FGFR1*, *FGFR2*, *FGFR3*, and *FGFR4* mRNA in PC9 and PC9/OR3 cells. **G** Western blot analysis of EGFR and FGFR1 signaling pathways in PC9 and PC9/OR3 cells following treatment with osimertinib for 24 h. **H** Cell proliferation assay of PC9 and PC9/OR3 cells following FGFR1 knockdown using siRNA for 5 days (left panel), and Western blot analysis of FGFR1 expression in PC9 and PC9/OR3 cells following FGFR1 knockdown using siRNA for 48 h (right panel). **I** Growth inhibition curves of PC9 and PC9/OR3 cells following treatment with FGFR1/2/3 inhibitor zoligratinib for 5 days. **J** Cell proliferation analysis was performed to assess the sensitivity of PC9 cells to osimertinib after treatment with osimertinib for 3 days following exogenous overexpression of FGFR1 (pCDNA3-FGFR1). **K** Western blot analysis of FGFR1/STAT3/PLK1 axis in PC9 and PC9/OR3 cells. **L** Western blot analysis of STAT3/PLK1 axis in PC9/OR3 cells following treatment with ponatinib for 24 h. **M** Western blot analysis of STAT3/PLK1 axis in PC9/OR3 cells following FGFR1 knockdown using siRNA for 48 h. (**N**) Western blot analysis of STAT3/PLK1 axis in PC9 cells following exogenous FGFR1 overexpression (pCDNA3-FGFR1) for 48 h. **O** RT-qPCR analysis of the *PLK1* mRNA expression in PC9 and PC9/OR3 cells. **P** RT-qPCR analysis of *PLK1* mRNA expression in PC9/OR3 cells following STAT3 knockdown using siRNA for 48 h. **Q** PC9 and PC9/OR3 cell lysates were immunoprecipitated with anti-PLK1 antibody and analyzed by Western blot analysis using the indicated antibodies, with normal rabbit IgG as a control. SE: short exposure; LE: long exposure. **R** Western blot analysis of PLK1/STAT3 axis in PC9/OR3 cells following PLK1 or STAT3 knockdown using siRNA for 48 h. **S** Cell proliferation analysis of PC9/OR3 cells following a 5-day treatment with osimertinib (3000 nM) after PLK1 or STAT3 knockdown using siRNA. ***P < 0.001.

As mentioned above, genes downregulated by volasertib might be associated with EGFR-TKI resistance. To explore this further, we established an osimertinib-resistant cell line (PC9/OR) by gradually increasing the concentration of osimertinib in the culture medium of PC9 cells over a period of approximately 10 months (Fig. 5A). Cellular sequencing analysis revealed no secondary mutations in the *EGFR* gene. Subsequently, we conducted RNA-seq analysis of both PC9 parental and resistant cells to elucidate the mechanisms underlying drug resistance. Transcriptomic profiling revealed significant transcriptomic alterations in PC9/OR cells compared to parental cells, with 1535 upregulated genes (Supplementary Fig. 4A). GO and KEGG enrichment analyses revealed that these upregulated genes were highly enriched in signaling pathways related to EMT and fibroblast growth factor receptor (FGFR) signaling (Fig. 5B and Supplementary Fig. 4B). Corresponding heatmap analysis further showed FGFR1 is involved in both the EMT and FGFR signaling pathways (Supplementary Fig. 4C).

To generate resistant cell lines with more stable characteristics, we isolated a series of PC9/OR-resistant clones through monoclonal screening (Fig. 5C). Western blot analysis revealed consistent downregulation of E-cadherin expression, alongside significant upregulation of N-cadherin and vimentin levels across the PC9/OR monoclonals, highlighting EMT as key characteristics associated with resistance (Fig. 5D). IC_50_ values showed that these PC9/OR monoclonals exhibited resistance not only to the third-generation EGFR-TKI osimertinib but also to first- and second-generation EGFR-TKIs (Fig. 5E and Supplementary Fig. 4D). Among these, the PC9/OR3 clone, exhibiting high resistance to various EGFR-TKIs including erlotinib, afatinib, and osimertinib, with respective resistance factors (RF) of 399, 5377, and 388 (Fig. 5E), was selected for further mechanistic investigation.

We then utilized RT-qPCR to analyze the mRNA expression levels of the FGFR family in PC9/OR3 cells, revealing significant upregulation of *FGFR1* without notable alterations in other FGFR family members (Fig. 5F). Consistently, Western blot analysis showed markedly higher FGFR1 expression in PC9/OR3 cells compared to parental cells, alongside reduced efficacy of osimertinib in suppressing EGFR downstream signaling (Fig. 5G). To investigate the role of FGFR1 in EGFR-TKI resistance, we knocked down FGFR1 using siRNA and found that the proliferation of PC9/OR3 cells was significantly inhibited, with a much greater effect than that in parental PC9 cells (Fig. 5H). Similarly, the FGFR1/2/3 inhibitor zoligratinib exhibited preferential antitumor activity against PC9/OR3 cells compared to parental counterparts (Fig. 5I). In contrast, FGFR1 overexpression in parental PC9 cells decreased sensitivity to osimertinib (Fig. 5J). These results underscore the critical role of aberrant FGFR1 signaling in EGFR-TKI resistance, consistent with previously reported findings [41, 42].

In addition, as illustrated in Supplementary Fig. 4E, 4F, transcriptomic analysis of GSE174850 data revealed significant upregulation of FGFR1 and PLK1 in osimertinib-resistant cell lines (R2) compared to non-resistant PC9-BrM4-control cell lines (C2), with a notable positive correlation between their expression. Together with our aforementioned findings that PLK1 and STAT3 are crucial regulators of EGFR-TKI sensitivity in NSCLC cells, we subsequently investigated their regulatory relationship with FGFR1 in EGFR-TKI-resistant cell lines. Western blot assays showed that concurrent upregulation of PLK1 expression and STAT3 phosphorylation in PC9/OR3 cells was consistent with elevated FGFR1 levels (Fig. 5K). Furthermore, the FGFR1 inhibitor ponatinib suppressed not only FGFR1 phosphorylation but also STAT3 phosphorylation and PLK1 expression (Fig. 5L). Consistently, siFGFR1-mediated knockdown in PC9/OR3 cells markedly reduced STAT3 phosphorylation and significantly decreased PLK1 expression (Fig. 5M). Conversely, forced overexpression of FGFR1 in parental PC9 cells resulted in concurrent upregulation of phosphorylated STAT3 and PLK1 expression (Fig. 5N). Together, these findings indicate that the FGFR1-mediated regulation of the STAT3/PLK1 axis is a key mechanism underlying resistance to EGFR-TKIs.

We next investigated the relationship between PLK1 and STAT3 in the EGFR-TKI-resistant cell line. RT-qPCR analysis revealed that *PLK1* mRNA expression in PC9/OR3 cells was significantly higher compared to that in parental PC9 cells (Fig. 5O) and attenuated by siRNA-mediated STAT3 silencing, consistent with findings observed in parental PC9 cells (Fig. 5P). Interestingly, co-IP experiments demonstrated a significantly stronger interaction between PLK1 and STAT3 in PC9/OR3 cells than that in parental PC9 cells (Fig. 5Q). Western blot analysis further revealed that siRNA-mediated PLK1 knockdown markedly reduced STAT3 phosphorylation, while STAT3 silencing downregulated PLK1 expression, confirming a STAT3-PLK1 positive feedback loop in PC9/OR3 cells, as in the parental PC9 cell line (Fig. 5R). Additionally, knockdown of either PLK1 or STAT3 significantly enhanced the sensitivity of PC9/OR3 cells to osimertinib (Fig. 5S). These findings indicate that the FGFR1-driven positive feedback loop between PLK1 and STAT3 plays a critical role in acquired resistance to EGFR-targeted therapies, and disrupting this regulatory axis may help overcome resistance to EGFR-TKIs.

### Targeting the PLK1/STAT3 axis enhances the efficacy of osimertinib in EGFR-TKIs- resistant models

**Fig. 6:**
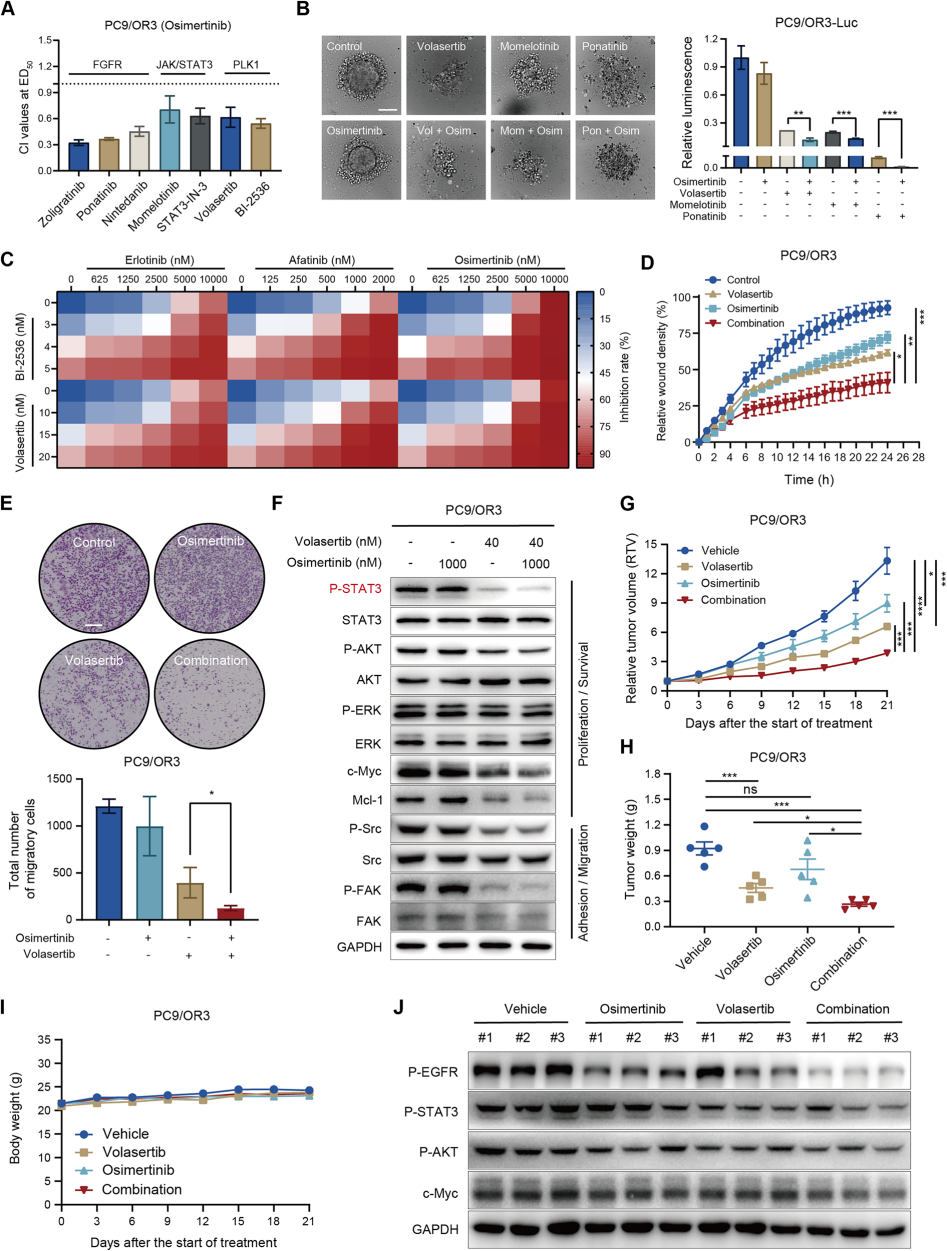
Targeting the PLK1/STAT3 axis enhances the efficacy of osimertinib in EGFR-TKIs- resistant models. **A** The CI values of osimertinib combined with FGFR/JAK/STAT3/PLK1 inhibitors in PC9/OR3 cells treated for 5 days, calculated using the Calcusyn software. **B** Representative image and quantitative luciferase analysis of PC9/OR3-Luc in the 3D culture system treated with osimertinib (1250 nM), ponatinib (250 nM), momelotinib (250 nM), volasertib (25 nM) or the combination for 8 days. Scale bar, 200 μm. **C** Cell proliferation analysis of PC9/OR3 cells following PLK1 inhibitors (volasertib and BI-2536), EGFR-TKIs (erlotinib, afatinib and osimertinib) alone or in combination for 5 days. **D** Wound healing assay of PC9/OR3 cell following osimertinib (5000 nM) combined with volasertib (50 nM) treatment (*P < 0.05, **P < 0.01, ***P < 0.001). **E** Transwell migration assay of PC9/OR3 cells following osimertinib (1000 nM) combined with volasertib (20 nM) treatment with for 24 h. Scale bar: 200 μm. **F** Western blot analysis of EGFR downstream signals (proliferation, survival, adhesion, and migration) in PC9/OR3 cells following osimertinib combined with volasertib treatment for 24 h. PC9/OR3 xenograft models were administered with vehicle, volasertib, osimertinib, or their combination, and relative tumor volume (**G**), tumor weight (**H**), and body weight (**I**) were monitored. Data represent the average and SEM (n = 5). Statistical significance was assessed one-way ANOVA (ns: no significance, *P < 0.05, ***P < 0.001, ****P < 0.0001). **J** Western blot analysis of key pathway proteins in tumor lysates.

Given the role of FGFR1 in promoting positive feedback loops between PLK1 and STAT3 (which contributes to EGFR-TKI resistance), we subsequently evaluated the effects of osimertinib combined with inhibitors targeting the FGFR/JAK/STAT3/PLK1 pathway in resistant cells. The results demonstrated that FGFR inhibitors (zoligratinib, ponatinib, nintedanib), JAK/STAT3 inhibitors (momelotinib, STAT3-IN-3), and PLK1 inhibitors (volasertib, BI-2536) significantly enhanced the antiproliferative effect of osimertinib in PC9/OR3 cells, with CI values < 1 (Fig. 6A). In three-dimensional (3D) culture models, osimertinib alone exhibited limited effects in PC9/OR3 cells even at high concentrations and with prolonged exposure, as evidenced by minimal changes in spheroid sizes and cell viability compared to the control (Fig. 6B). In contrast, monotherapy with the PLK1 inhibitor volasertib, the JAK1/2 inhibitor momelotinib, or the FGFR inhibitor ponatinib significantly reduced spheroid size and viability in PC9/OR3 cells, with a further enhanced inhibitory effect when combined with osimertinib (Fig. 6B). Additionally, PLK1 inhibitors (BI-2536 and volasertib) significantly enhanced the sensitivity of PC9/OR3 cells to various EGFR-TKIs, including erlotinib, afatinib, and osimertinib (Fig. 6C).

Considering the established link between EMT, tumor metastasis, and poor prognosis in EGFR-mutant NSCLC, we investigated the effects of PLK1 inhibitors combined with osimertinib on the migratory properties of resistant cells. Our results showed that this combination treatment significantly reduced the migratory capacity of PC9/OR3 cells compared to volasertib or osimertinib monotherapy, as evidenced by real-time wound healing analysis (Fig. 6D) and transwell assays (Fig. 6E). Mechanistically, the combination treatment enhanced the inhibition of STAT3 and AKT phosphorylation, downregulated the expression of c-Myc and Mcl-1, and inhibited the phosphorylation of Src and FAK—key mediators of cell migration (Fig. 6F).

To validate the therapeutic potential of this combination strategy, the antitumor efficacy of volasertib combined with osimertinib was further assessed in the PC9/OR3 xenograft model. Consistent with our in vitro findings, osimertinib monotherapy at 2.5 mg/kg demonstrated limited efficacy in PC9/OR3 xenograft models, while volasertib at 20 mg/kg exhibited moderate antitumor activity with efficacy comparable to that observed in the parental model. Notably, the combination treatment showed more pronounced suppression of tumor growth (Fig. 6G) and a substantial reduction in tumor weight compared to the monotherapy groups (Fig. 6H). Moreover, all treatment regimens were well tolerated, and no significant body weight changes were observed during the experiment (Fig. 6I). Western blot analysis of tumor tissues revealed significant inhibition of STAT3 and AKT phosphorylation, as well as downregulation of c-Myc expression, in the combination group (Fig. 6J). Collectively, these results indicate that targeting the PLK1/STAT3 axis not only enhances the therapeutic efficacy of EGFR/MEK/ERK pathway inhibitors but also represents a promising strategy to delay and overcome resistance in EGFR-mutant NSCLC.

## Discussion

**Fig. 7:**
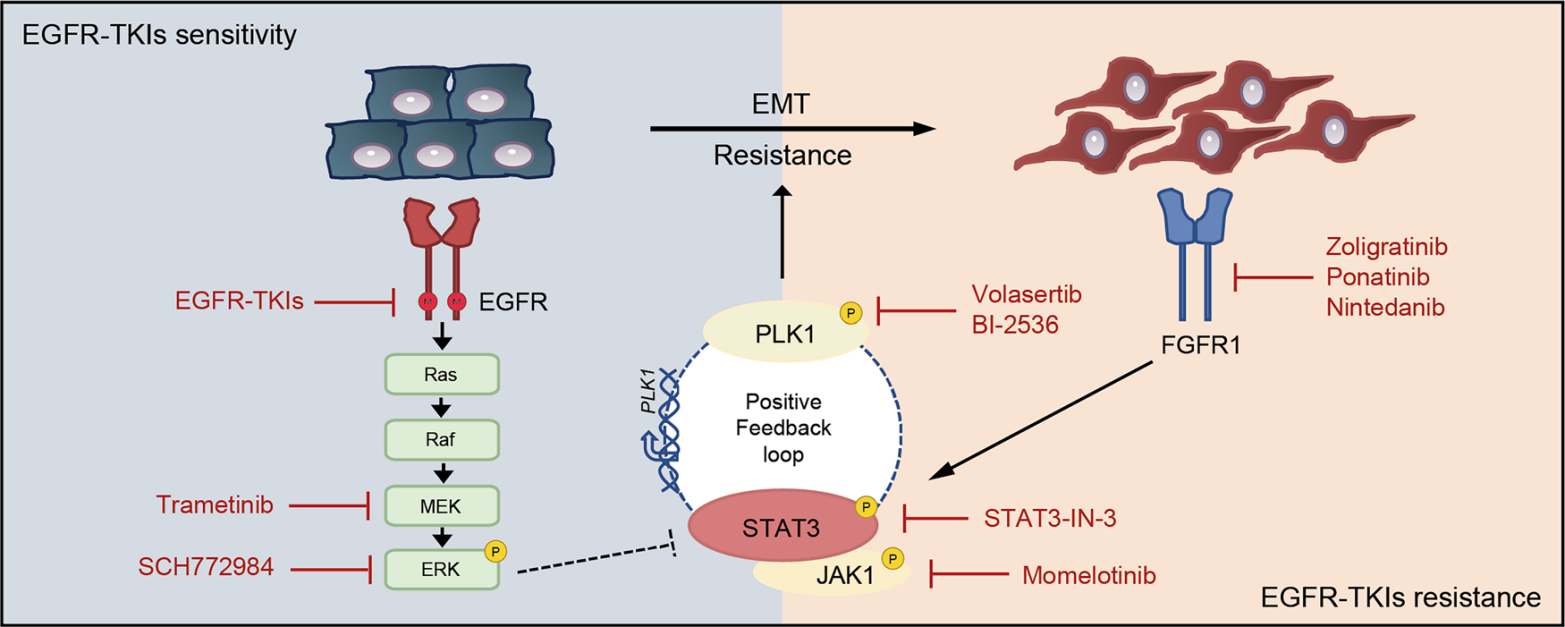
Schematic diagram illustrating that co-targeting FGFR1/STAT3/PLK1 and the EGFR/MAPK signaling pathway enhances efficacy and delays FGFR1-driven resistance onset in EGFR-mutant NSCLC. Created with BioRender.com.

Patients with advanced NSCLC harboring EGFR mutations typically exhibit favorable initial responses to EGFR-TKIs. However, therapeutic resistance inevitably develops, often due to secondary EGFR mutations or alternative non-EGFR-independent mechanisms, such as EMT and activation of bypass signaling pathways [6]. This persistent therapeutic challenge underscores the critical unmet clinical need in managing EGFR-mutant NSCLC and highlights the urgent necessity of developing innovative combination strategies. To address this challenge, we conducted high-throughput drug screening using two representative EGFR-mutant NSCLC cell lines—PC9 (EGFR exon 19 ELREA-Del) and NCI-H1975 (EGFR L858R/T790M). This screening identified several promising therapeutic targets, with PLK1 emerging as a particularly strong candidate. Our results indicated that PLK1 inhibitors, such as volasertib, exhibited broad cytotoxicity across various EGFR-mutant cell lines, including those harboring secondary mutations associated with resistance. The combination of EGFR-TKIs and PLK1 inhibitors showed synergistic effects, effectively overcoming EGFR-TKI resistance both in vitro and in vivo. Mechanistically, PLK1 inhibitors reversed the activation of STAT3, which was initially triggered by both EGFR-TKIs and MEK/ERK inhibitors, and suppressed the subsequent transcriptional upregulation of its downstream target genes in EGFR-mutant NSCLC cell lines. Furthermore, we identified a positive feedback loop between PLK1 and STAT3 that progressively accelerates the development of acquired resistance to EGFR-TKIs, leading to increased reliance on FGFR1 signaling and subsequent induction of EMT (Fig. 7). Therefore, simultaneous inhibition of the EGFR/MEK/ERK pathway and disruption of the PLK1-STAT3 axis effectively enhance antitumor efficacy and delay the development of acquired resistance in EGFR-mutant NSCLC.

PLK1, a critical oncogenic kinase overexpressed in various cancers, has emerged as a promising therapeutic target, with several inhibitors (e.g., BI-2536) that previously underwent clinical trials for solid tumors, including NSCLC [30, 31, 33, 35, 43]. However, the precise mechanism underlying PLK1’s role in tumorigenesis and its functional significance in EGFR-mutant NSCLC remain incompletely understood [34]. Analysis of clinical databases revealed that *PLK1* mRNA expression is more markedly elevated in tumor tissues than in adjacent normal tissues from EGFR-mutant NSCLC patients. Furthermore, high *PLK1* mRNA expression not only correlated with additional mutations in ErbB signaling pathway components (beyond primary EGFR mutations) but also served as an unfavorable prognostic indicator in this patient population. In other words, multiple factors must be considered in managing EGFR-mutant NSCLC, including the limitations of EGFR-TKI monotherapy, the co-occurrence of other genetic alterations, and the prognostic implication of high PLK1 expression. Supporting this approach, previous studies [44–46] and KEGG pathway enrichment analysis of RNA-seq data from PLK1 inhibitor-treated EGFR-mutant NSCLC cells demonstrated that PLK1 inhibition predominantly affects several critical signaling pathways, including those involved in apoptosis and EGFR-TKI resistance mechanisms. The distinct molecular characteristics and clinical significance of PLK1 overexpression provide an optimal framework for investigating both its mechanistic role and potential therapeutic synergies. Accordingly, our previous study showed that the PLK1/c-Myc pathway mediates resistance to KRAS^G12C^ inhibitors in NSCLC, and co-inhibition of PLK1 and KRAS^G12C^ synergistically suppresses tumor growth and overcomes this resistance [47].

The clinical development of PLK1 inhibitors for NSCLC faces challenges due to their narrow therapeutic window and dose-limiting toxicity, highlighting the need for therapeutic index optimization and a better understanding of the mechanisms of PLK1 inhibition to advance effective combination therapies [29]. PLK1 has emerged as a key regulator of multiple transcription factors, including STAT3, c-Myc, FOXM1, and β-catenin, positioning it as a promising combinatorial therapeutic target for disrupting oncogenic signaling networks in cancer [24, 27, 47]. Regarding the regulatory relationship between PLK1 and STAT3, our study provides novel mechanistic insights, demonstrating that PLK1 regulates STAT3 activity by engaging in protein-protein interactions and facilitating JAK1-mediated phosphorylation, although the binding sites of PLK1 with JAK/STAT3 and the precise molecular mechanisms require additional investigation. This regulatory relationship is further reinforced by STAT3-mediated transcription of PLK1, establishing a robust positive feedback loop that not only reduces sensitivity to EGFR-TKIs in EGFR-mutant NSCLC but also significantly impacts patient prognosis. These findings position PLK1 inhibitors as promising therapeutic agents, particularly when combined with targeted therapies that induce STAT3 activation, including EGFR/MEK/ERK inhibitors in EGFR-mutant NSCLC. While previous literature indicated that PLK1 modulates STAT3 transcription via β-catenin in esophageal cancer cell lines [40], our study acknowledges that additional factors may contribute to the PLK1/STAT3 axis, warranting further investigation into the complex molecular mechanisms underlying this signaling network. Future research should focus on identifying additional components of this regulatory axis and exploring optimal combination strategies to maximize therapeutic efficacy while minimize toxicity.

Constitutive activation of STAT3 is frequently observed across malignancies, which can be attributed to activating mutations, excessive signaling from upstream regulators, or impaired negative feedback regulation [48]. Emerging evidence highlights that upregulation of transcription factors such as STAT3 and c-Myc represents a activation-mediated mechanism by which tumors evade targeted therapies, particularly small-molecule inhibitors [15, 49]. In light of our findings and the published literature [15], we postulated that the activation of STAT3 serves as a “self-protective” mechanism employed by tumor cells to counteract inhibition of the EGFR/MEK/ERK pathway. The protective effect of STAT3 activation on tumor cell apoptosis following drug treatment is closely associated with the regulation of specific genes [48]. Historically considered an “undruggable” target, STAT3 currently has no specific targeting drugs available [50], but several inhibitors and PROTACs (e.g., KT-333) have demonstrated promising antitumor activity in preclinical and clinical trials [51, 52]. These findings further suggest that targeting transcription factors such as STAT3 has the potential to revolutionize the pharmaceutical landscape and provide improved options for combination therapy strategies in EGFR-mutant NSCLC patients.

The management of NSCLC continues to face significant challenges due to tumor recurrence and metastasis, which are mediated by complex mechanisms involving the activation of bypass signaling pathways and phenotypic transformation [53]. This multifaceted process evolves gradually through intricate molecular interactions and compensatory mechanisms rather than occurring as an abrupt event [54]. In response to these challenges, combination therapies incorporating EGFR-TKIs are being investigated in patients with early-stage or locally advanced EGFR-mutant NSCLC to achieve durable remission [53]. Our preclinical study found that the combination of the PLK1 inhibitor volasertib with the EGFR-TKI osimertinib induced complete tumor regression in EGFR-mutant NSCLC xenograft models, with sustained effects observed even after treatment cessation. This indicates the potential for developing durable therapeutic strategies that promote long-term tumor control and reduce recurrence risk in clinical settings. Although our in vivo model of EGFR-TKI resistance showed that the combination of osimertinib and volasertib demonstrated enhanced efficacy compared to monotherapy, it failed to achieve tumor regression. Mechanistic investigations further revealed that the positive feedback loop within the FGFR1-driven PLK1/STAT3 signaling pathway serves as a critical regulatory node mediating acquired resistance to EGFR-TKI therapy in NSCLC. These results indicate that FGFR1 activation is essential for the development of tolerance to the combination of osimertinib and volasertib in EGFR-mutant NSCLC cells, highlighting FGFR1 signaling as a critical feedback-mediated mechanism that warrants further investigation. This observation aligns with previous studies demonstrating that combining osimertinib with FGFR inhibitors overcomes resistance mediated by abnormal FGFR expression [55]. Emerging evidence suggests that multi-targeted combination therapies incorporating three or more molecular targeted agents (including EGFR-TKIs) may represent a promising strategy to prevent or delay the development of acquired resistance in EGFR-mutated NSCLC [56, 57]. Ryota Nakamura et al. [58]. reported promising results from triple combination therapy using osimertinib, AXL inhibitors, and FGFR inhibitors in EGFR-mutant NSCLC. Moving forward, we propose exploring a three-drug combination regimen involving FGFR inhibitors, osimertinib, and PLK1 inhibitors, which might more effectively eradicate residual tumor cells and promote tumor regression. Importantly, our current study does not rule out the potential involvement of additional resistance mechanisms to EGFR-TKIs, underscoring the necessity of comprehensive investigations to fully elucidate the molecular determinants of treatment response and resistance in EGFR-mutant NSCLC.

Another particularly pressing clinical challenge is the high incidence of brain metastases in EGFR-mutant lung cancer patients, which remains difficult to manage effectively [59, 60]. The clinical management of EGFR-mutant NSCLC faces significant challenges, including the limited efficacy of osimertinib monotherapy in controlling brain metastases and the inevitable development of acquired resistance [61, 62]. Notably, PLK1 inhibitors (e.g., volasertib and GSK461364A) have demonstrated blood-brain barrier penetration capabilities [63], suggesting their potential utility in combination with osimertinib to address these therapeutic challenges. While the current study did not specifically investigate brain metastasis models, these findings provide valuable insights and lay the groundwork for future research in this critical area.

In summary, our research identifies the PLK1/STAT3 axis as a crucial driver and vulnerability in both EGFR-TKI-sensitive and -resistant tumors, highlighting the potential of targeting PLK1 to address this challenge. Our preclinical study demonstrates that combining PLK1 inhibitors with EGFR/MEK/ERK pathway inhibitors not only exhibits remarkable efficacy in eradicating cancer cells but also effectively overcomes the acquired resistance to EGFR-TKIs, thus offering a potential clinical treatment strategy for EGFR-mutant NSCLC patients.

## Supplementary information

Supplementary material

Original Western Blots

## Data Availability

Immunohistochemical data were sourced from the Human Protein Atlas database (HPA, https://www.proteinatlas.org/). The Cancer Genome Atlas (TCGA, https://portal.gdc.cancer.gov/) was used to retrieve the gene expression, mutation, and clinical information of the NSCLC patients. The correlation between PLK1 and EGFR expression was analyzed using Gene Expression Profiling Interactive Analysis (GEPIA, http://gepia.cancer-pku.cn/) database. *FGFR1* and *PLK1* expression data were obtained from GSE174850 in the Gene Expression Omnibus database (GEO, https://www.ncbi.nlm.nih.gov/geo/).
