# Supplementary material for: Targeting PLK1 potentiates the antitumor efficacy of EGFR-TKIs through inhibiting the JAK1/STAT3 pathway
Source: Cell Death Dis. 2026 Jan 15;17(1):41. doi: 10.1038/s41419-025-08220-9 (PMC12808168; doi:10.1038/s41419-025-08220-9)
Supplement: Supplementary file 1 — Supplementary material [file 41419_2025_8220_MOESM1_ESM.pdf]

SUPPLEMENTARY MATERIAL

Targeting PLK1 potentiates the antitumor efficacy of EGFR-TKIs through inhibiting the JAK1/STAT3 pathway

Cheng Li[1, 2], Shangxuan Shi[1, 2], Long Li[2], Yafang Wang[2], Mingyue Yao[3], Chengcheng Yu[3], Chuwei Yu[3], Chengying Xie[1, 2, 3]*

[1]School of Life Science and Technology, ShanghaiTech University, 393 Middle Huaxia Road, Shanghai 201210, China;

[2]Shanghai Institute for Advanced Immunochemical Studies, ShanghaiTech University, 393 Middle Huaxia Road, Shanghai 201210, China;

[3]Lingang Laboratory, 2380 Hechuan Road, Shanghai 201101, China.

***Corresponding authors:**

Chengying Xie, Lingang Laboratory, 2380 Hechuan Road, Shanghai 201101, China, E-mail: xiecy@lglab.ac.cn. Tel/Fax: 86-021-58156326.

SUPPLEMENTARY FIGURES

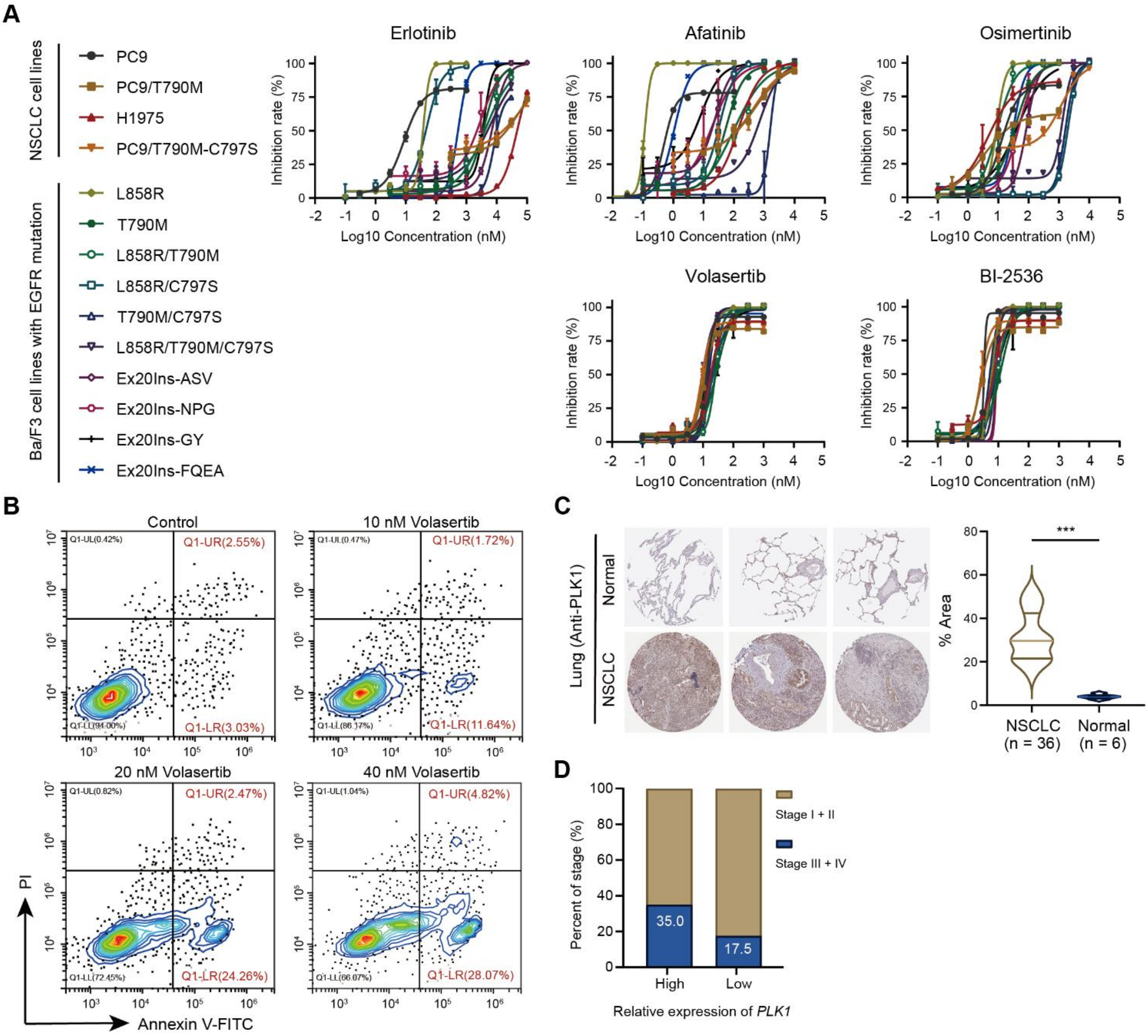

Supplementary Fig. 1 PLK1 inhibitors promise for the treatment of EGFR-mutant NSCLC.

(A) Growth inhibition curves of EGFR-mutant NSCLC and Ba/F3 cell lines harboring various EGFR mutations following EGFR-TKIs and PLK1 inhibitors treatment for 5 days. **(B)** The representative flow cytometry plots illustrating the effects of volasertib on apoptosis in PC9 cells were assessed using annexin V/PI staining. **(C)** Immunohistochemical (IHC) analysis of PLK1 expression, including adenocarcinoma and squamous cell carcinoma, as well as in normal tissues, was conducted using data from the Human Protein Atlas (HPA) database. The violin plot was generated through quantitative

analysis of IHC data using Image J (***$P < 0.001$). **(D)** The distribution of patients across different stages was compared between the high PLK1 expression group and the low PLK1 expression group.

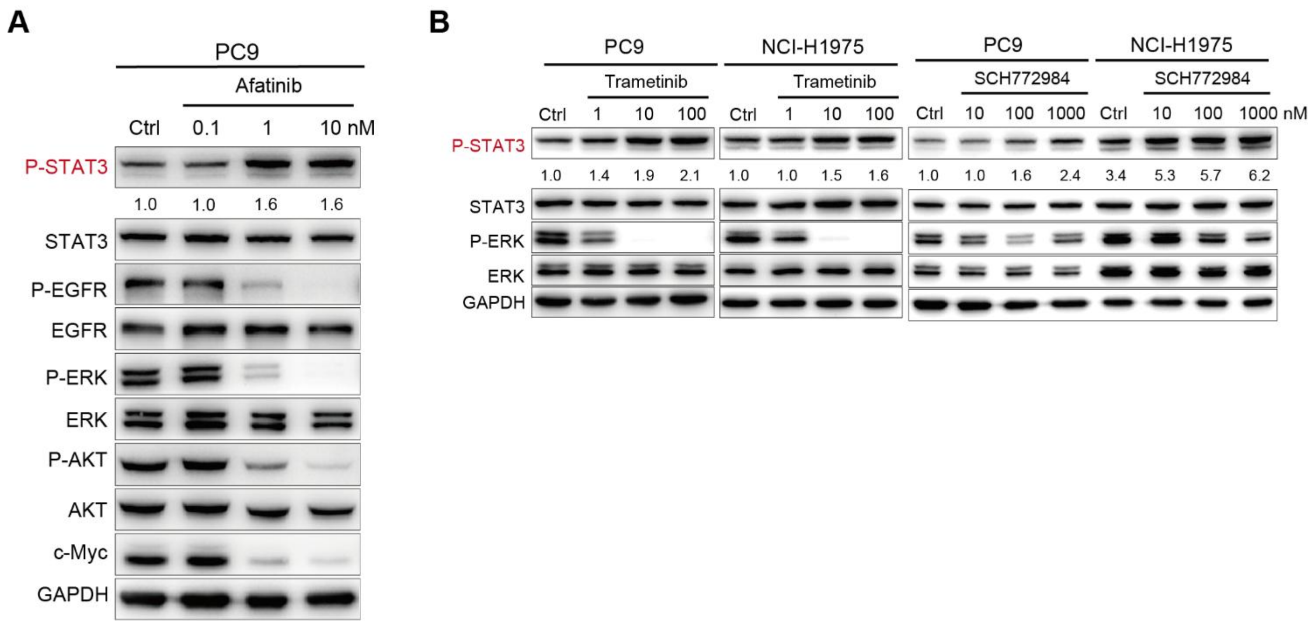

Supplementary Fig. 2 EGFR/MEK/ERK inhibitors induce activation of STAT3 in EGFR-mutated NSCLC cell lines.

(A) Western blot analysis of EGFR signaling pathways in PC9 and NCI-H1975 cells following treatment with afatinib for 24 h. **(B)** Western blot analysis of STAT3 phosphorylation in PC9 and NCI-H1975 cells following treatment with the MEK inhibitor trametinib or the ERK inhibitor SCH772984 for 24 h.

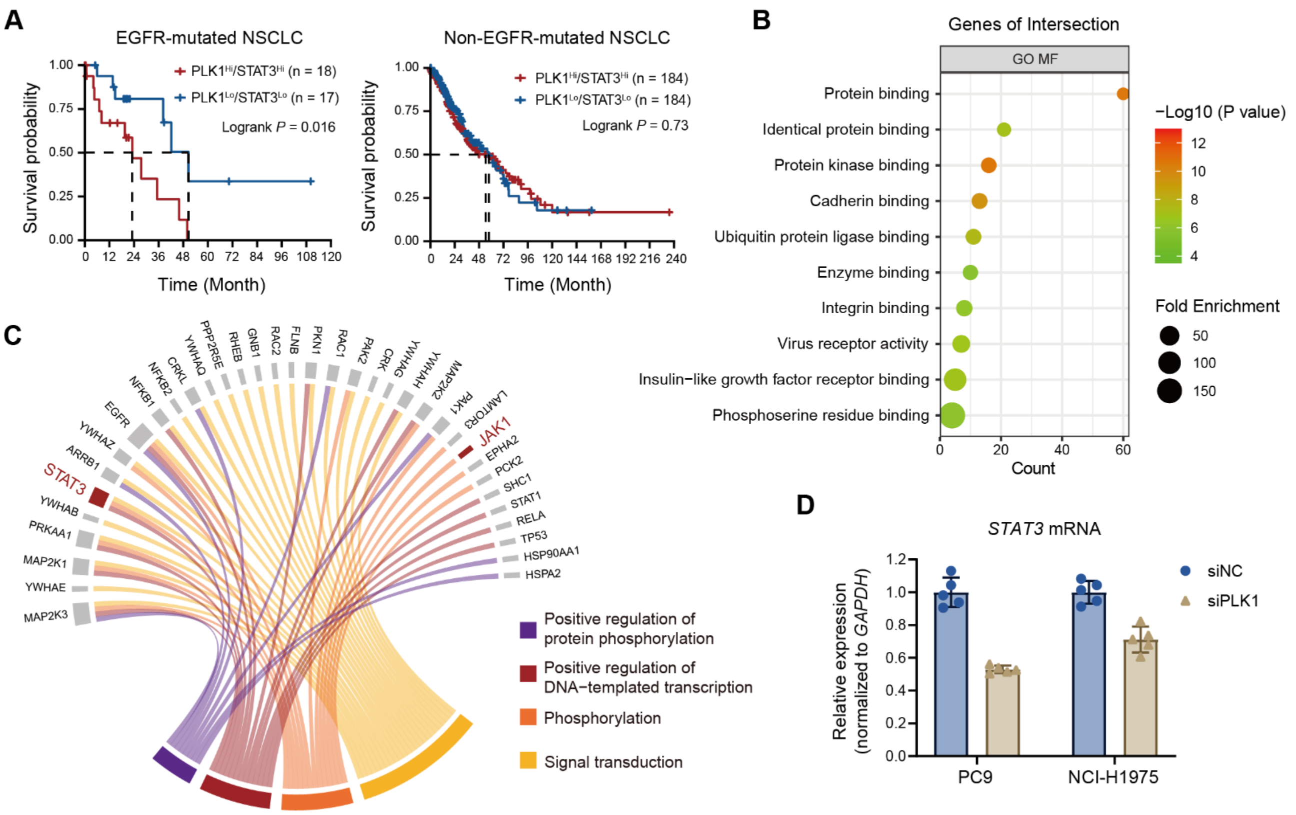

Supplementary Fig. 3 PLK1 contributes to the JAK1-mediated phosphorylation of STAT3.

(A) Kaplan-Meier survival curves were constructed to assess the effect of PLK1/STAT3 co-overexpression on the prognosis of EGFR-mutant or non-EGFR mutant NSCLC patients. **(B)** GO/MF enrichment analysis of 60 overlapping proteins ($P < 0.05$, Top10). **(C)** The chord diagram analysis of the relationships between JAK1/STAT3 and signal transduction, phosphorylation, and transcription-related biological functions. **(D)** RT-qPCR analysis of *STAT3* mRNA expression in PC9 and NCI-H1975 cells following PLK1 knockdown using siRNA for 48 h.

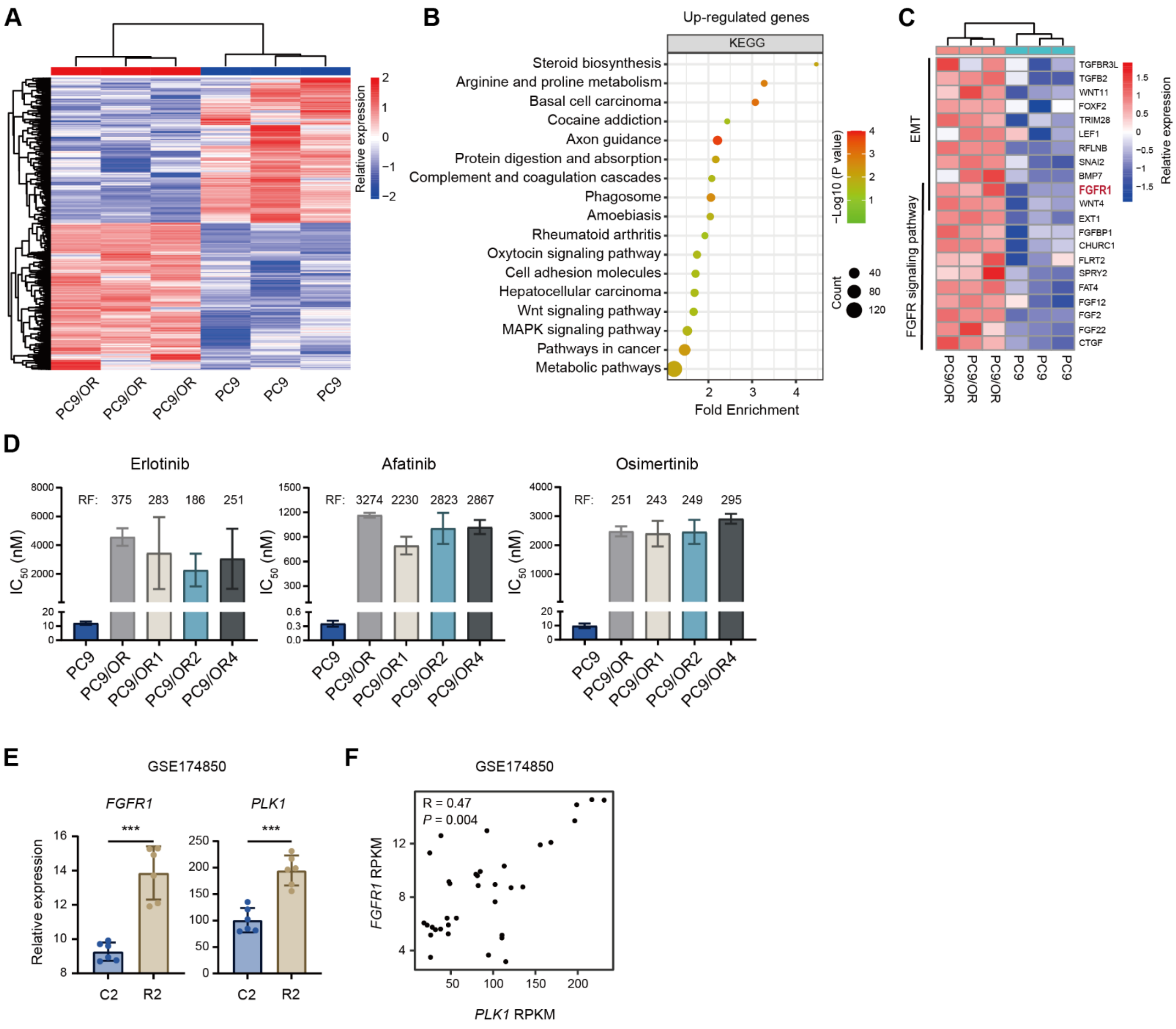

Supplementary Fig. 4 Abnormal expression of FGFR1 mediates resistance to EGFR-TKIs in EGFR-mutated NSCLC.

(A) The hierarchical clustering and heatmap analysis of differentially expressed genes (DEGs) in parental PC9 cells and resistant PC9/OR cells. **(B)** KEGG enrichment analysis of up-regulated genes in PC9/OR cells compared to PC9 cells, (P < 0.05, Top20). **(C)** Heatmap analysis of *FGFR1* enrichment in the EMT and FGFR signaling pathways. **(D)** IC_{50} values of EGFR-TKIs in PC9 and PC9/OR, PC9/OR1, PC9/OR2, PC9/OR4 cells. The resistance factor (RF) was calculated as the ratio of IC50 values (resistant line/parental line). **(E-F)** Data from GSE174850 in the GEO database were utilized. **(E)** Relative expression levels of *FGFR1* and *PLK1* were measured in PC9-BrM4-Control-2

(C2) and PC9-BrM4-Osimertinib-Resistance-2 (R2) samples (***$P < 0.001$). **(F)** The correlation between *FGFR1* and *PLK1* mRNA expression was examined in brain metastatic outgrowth and osimertinib resistant cells.

SUPPLEMENTARY TABLES

Supplementary Table S1. Cell proliferation inhibition rates of PC9 and NCI-H1975 cells following different compound treatments for 5 days.

Number	Compounds	Target Protein	Inhibition rate (%, 100 nM)	
			PC9	NCI-H1975
1	Erlotinib	EGFR	74.1 ± 6.2	-8.0 ± 8.2
2	Afatinib	EGFR, HER2	76.4 ± 3.7	43.0 ± 2.6
3	Osimertinib	EGFR	78.3 ± 2.8	82.9 ± 2.0
4	Volasertib	PLK1	88.8 ± 6.0	84.1 ± 4.7
5	BI-2536	PLK1, BRD4	85.0 ± 8.9	82.6 ± 6.9
6	Ponatinib	FGFR1, Src, ABL, PDGFRα, VEGFR2	45.0 ± 0.3	17.5 ± 0.9
7	Nintedanib	FGFR1/2/3, VEGFR1/2/3, PDGFRα/β	15.2 ± 4.1	1.9 ± 3.5
8	Suntinib	VEGFR2, PDGFRβ	36.2 ± 5.1	32.6 ± 1.0
9	Sorafenib	Raf, VEGFR2, VEGFR3, PDGFRβ, FLT3, c-Kit	6.0 ± 3.4	-3.4 ± 11.1
10	BAY-293	SOS1	49.1 ± 0.8	44.0 ± 2.5
11	BI-3406	SOS1	27.0 ± 0.9	9.1 ± 12.0
12	ARS-1620	$KRAS^{G12C}$	39.9 ± 1.9	27.5 ± 2.0
13	AMG-510	$KRAS^{G12C}$	39.0 ± 2.5	24.2 ± 2.0
14	MRTX-849	$KRAS^{G12C}$	-3.3 ± 1.6	-22.0 ± 4.1
15	MRTX-1133	$KRAS^{G12D}$	42.3 ± 1.0	30.6 ± 2.5
16	RMC-6236	RAS $(ON)^{MULTI}$	71.1 ± 1.4	52.0 ± 7.9
17	Trametinib	MEK1/2	77.2 ± 8.1	84.2 ± 1.2
18	SCH772984	ERK1/2	65.2 ± 2.0	36.3 ± 4.9
19	Dasatinib	BCR-ABL, Src	71.7 ± 2.5	59.5 ± 6.4
20	Imatinib	BCR/ABL, v-ABL, PDGFR, c-kit	48.3 ± 3.6	42.3 ± 0.1
21	Nilotinib	BCR-ABL	43.9 ± 3.5	43.5 ± 0.0
22	Olverembatinib	pan-BCR-ABL	65.6 ± 2.6	79.1 ± 1.1
23	Momelotinib	JAK1/2	48.0 ± 2.6	33.1 ± 17.5
24	STAT3-IN-3	STAT3	15.7 ± 16.2	11.1 ± 12.3
25	GDC-0941	PI3Kα/δ	26.3 ± 3.4	36.5 ± 6.6
26	BKM-120	pan-class I PI3K	14.8 ± 3.0	9.4 ± 8.1
27	CAL-101	p110δ	42.7 ± 3.2	34.6 ±4.1
28	MYCi975	MYC	3.2 ± 6.0	0.3 ± 4.8
29	AC220	FLT3	49.1 ± 0.7	46.0 ± 3.1
30	Gilteritinib	FLT3, AXL	45.2 ± 2.5	8.0 ± 2.1
31	Crenolanib	FLT3, PDGFRα/β	22.1 ± 2.3	23.8 ± 2.3
32	Ibrutinib	BTK	52.4 ± 0.1	30.8 ± 6.9
33	Acalabrutinib	BTK	2.2 ± 5.3	-3.1 ± 12.2
34	Zanubrutinib	BTK	4.0 ± 3.1	-21.2 ± 9.7
35	BI-3802	BCL6	25.5 ± 0.2	14.3 ± 7.3
36	ARV-393	BCL6	33.1 ± 2.6	31.3 ± 1.4
37	AT7519	CDK	42.8 ± 1.9	63.0 ± 2.3

(Continued) Supplementary Table S1. Cell proliferation inhibition rates of PC9 and NCI-H1975 cells following different compound treatments for 5 days.

Number	Compounds	Target Protein	Inhibition rate (%, 100 nM)	
			PC9	NCI-H1975
38	BAY-1251152	CDK9	27.5 ± 3.3	39.8 ± 4.7
39	LDC000067	CDK9	46.6 ± 3.2	40.3 ± 0.5
40	AZD-4573	CDK9	97.0 ± 0.5	96.3 ± 0.4
41	Atuveciclib	CDK9, PTEFb	42.4 ± 1.5	43.7 ± 2.3
42	S63845	MCl-1	34.3 ± 1.7	36.4 ± 7.1
43	ABT-199	Bcl-2	43.1 ± 2.2	35.9 ± 2.4
44	MS-275	HDAC1, HDAC2, HDAC3	50.7 ± 2.8	65.2 ± 5.1
45	MGCD0103	HDAC1, HDAC2, HDAC3, HDAC11	60.5 ± 1.5	76.4 ± 2.1
46	APR-246	p53	34.1 ± 3.0	29.4 ± 0.5
47	Rezatapopt	p53	10.5 ± 4.5	11.7 ± 5.3
48	BAY 11-7082	IκBα, NF-κB	33.8 ± 3.5	33.2 ± 2.5
49	Lenalidomide	IKZF1/3	13.6 ± 2.6	3.6 ± 13.7
50	A-485	p300/CBP	5.9 ± 2.5	16.2 ± 3.5

Notes: all compounds were dissolved in DMSO at 10 mM stock solution and stored at -80°C.

Supplementary Table S2. The information of proteins interacting with PLK1 as identified by LC-MS/MS (60 proteins that intersect with ErbB signaling pathways including MAPK, PI3K/AKT, JAK/STAT).

Number	Gene names	Unique peptides	Score	Mean intensity
1	HSP90AB1	18	205.52	8402100000
2	HSPA8	19	278.06	6829200000
3	HSPB1	11	119.42	6553200000
4	HSP90AA1	19	103.37	4251000000
5	YWHAZ	9	25.76	1980100000
6	FLNA	40	140.98	1691300000
7	YWHAE	7	27.785	1022600000
8	RPS6	7	15.807	813220000
9	HSP90B1	14	20.537	695010000
10	PPP2R1A	13	42.438	524850000
11	GNB2	3	36.959	369410000
12	CDC42	2	12.099	247700000
13	FLNB	14	24.522	230830000
14	YWHAG	5	8.3875	210730000
15	YWHAQ	6	11.2	172940000
16	YWHAH	7	18.039	162920000
17	YWHAB	4	6.2558	140980000
18	CDC37	2	4.367	123550000
19	ITGB1	4	6.236	122220000
20	MAP2K2	4	3.9657	98694000
21	RAC2	2	8.7398	92339000
22	PAK2	4	5.9252	78574000
23	STAT1	5	7.0699	71229000
24	STAT3	6	12.311	69576000
25	RHEB	3	6.3321	48012000
26	TNC	4	10.316	44116000
27	CRKL	3	9.7995	42125000
28	RRAS	1	8.0212	42088000
29	ITGAV	5	20.52	41430000
30	TP53	4	2.7951	37677000
31	RAC1	2	1.3474	34616000
32	ITGA3	2	3.2681	25096000
33	ITGA5	2	2.5832	24917000
34	GNB1	2	6.5816	24351000
35	ITGA2	3	3.4766	21150000
36	MAP2K1	2	0.80389	15348000
37	NFKB2	2	3.8911	14746000
38	DUSP3	1	2.3951	13544000

(Continued) Supplementary Table S2. The information of proteins interacting with PLK1 as identified by LC-MS/MS (60 proteins that intersect with ErbB signaling pathways including MAPK, PI3K/AKT, JAK/STAT).

Number	Gene names	Unique peptides	Score	Mean intensity
39	EGFR	1	2.0991	13502000
40	PPP2R5E	2	1.4443	11285000
41	PRKAA1	1	1.3193	11285000
42	LAMB3	1	1.4678	11039000
43	JAK1	2	1.9806	8780000
44	PPP5C	1	2.3158	8453300
45	NFKB1	1	0.93029	8205400
46	MAP2K3	1	0.95602	7439400
47	EPHA2	1	0.87545	7374700
48	PKN1	1	0.60437	7256200
49	SHC1	1	0.72737	6861900
50	PAK1	1	0.95262	6488300
51	PCK2	1	1.4468	6387300
52	HSPA2	1	0.64089	5169600
53	RRAS2	1	1.1493	5112900
54	RELA	1	4.0504	4659300
55	EIF4B	1	0.91293	3892700
56	CRK	1	1.5244	3364700
57	ITGB5	1	0.70623	3276800
58	ARRB1	1	1.74	3004600
59	GYS1	1	0.97353	2493300
60	LAMTOR3	1	0.82071	0

Supplementary Table S3. Source of antibodies.

Antibodies (name in article)	Source	Identifier
EGF Receptor (D38B1) XP® Rabbit mAb (EGFR)	Cell Signaling Technology (CST)	Cat#: 4267
Phospho-EGF Receptor (Tyr1068) (D7A5) XP® Rabbit mAb (P-EGFR)	CST	Cat#: 3777
PLK1 (208G4) Rabbit mAb (PLK1)	CST	Cat#: 4513
PLK1 Antibody (13E8) (PLK1)	Novus	Cat#: NB100-74502
Stat3 (D3Z2G) Rabbit mAb (STAT3)	CST	Cat#: 12640
Phospho-Stat3 (Tyr705) (D3A7) XP® Rabbit mAb (P-STAT3)	CST	Cat#: 9145
Anti-STAT3 (phospho Y705) ((P-STAT3)	abcam	Cat#: ab76315
Akt (pan) (C67E7) Rabbit mAb (AKT)	CST	Cat#: 4691
Phospho-Akt (Ser473) (D9E) XP® Rabbit mAb (P-AKT)	CST	Cat#: 4060
p44/42 MAPK (Erk1/2) (137F5) Rabbit mAb (ERK)	CST	Cat#: 4695
Phospho-p44/42 MAPK (Erk1/2) (Thr202/Tyr204) (D13.14.4E) XP® Rabbit mAb (P-ERK)	CST	Cat#: 4370
c-Myc (E5Q6W) Rabbit mAb (c-Myc)	CST	Cat#: 18583
Mcl-1 (D2W9E) Rabbit mAb (Mcl-1)	CST	Cat#: 94296
PARP (46D11) Rabbit mAb (PARP/Cleaved PARP)	CST	Cat#: 9532
Caspase-3 (D3R6Y) Rabbit mAb (Caspase-3)	CST	Cat#: 14220
Cleaved Caspase-3 (Asp175) (5A1E) Rabbit mAb (Cleaved caspase-3)	CST	Cat#: 9664
Src (36D10) Rabbit mAb (Src)	CST	Cat#: 2109
Phospho-Src-Y419 Rabbit mAb (P-Src)	ABclonal	Cat#: AP1027
Jak1 (E3A6M) Rabbit mAb (JAK1)	CST	Cat#: 29261
Phospho-Jak1(Tyr1034/1035) (D7N4Z) Rabbit mAb (P-JAK1)	CST	Cat#: 74129
E-Cadherin (24E10) Rabbit mAb (E-cadherin)	CST	Cat#: 3195
N-Cadherin (D4R1H) XP® Rabbit mAb (N-cadherin)	CST	Cat#: 13116
Vimentin (D21H3) XP® Rabbit mAb (Vimentin)	CST	Cat#: 5741
FGF Receptor 1 (D8E4) XP® Rabbit mAb (FGFR1)	CST	Cat#: 9740
Phospho-FGFR1-Y653/Y654 Rabbit pAb (P-FGFR1)	ABclonal	Cat#: AP1317
FAK (D2R2E) Rabbit mAb (FAK)	CST	Cat#: 13009
Phospho-FAK (Tyr397) (D20B1) Rabbit mAb (P-FAK)	CST	Cat#: 8556
Histone H3 (D1H2) XP® Rabbit mAb (H3)	CST	Cat#: 4499
GAPDH (14C10) Rabbit mAb (GAPDH)	CST	Cat#: 2118
Goat Anti-Mouse IgG, H&L Chain Specific Peroxidase Conjugate	Merck Millipore	Cat#: 401215
Goat Anti-Rabbit IgG, H & L Chain Specific Peroxidase Conjugate	Merck Millipore	Cat#: 401353
Alexa Fluor™ 633 goat anti-mouse IgG (H+L)	Thermo Fisher Scientific	Cat#: 1845042
Alexa Fluor™ 488 goat anti-rabbit IgG (H+L)	Thermo Fisher Scientific	Cat#: 1853312

Supplementary Table S4. Primer sequence information used in the RT-qPCR assay.

Primer name	Forward primer (5' to 3')	Reverse primer (5' to 3')
GAPDH	GGAGCGAGATCCCTCCAAAAT	GGCTGTTGTCATACTTCTCATGG
PLK1	AAAGAGATCCCGGAGGTCCTA	GGCTGCGGTGAATGGATATTTC
STAT3	CAGCAGCTTGACACACGGTA	AAACACCAAAGTGGCATGTGA
FGFR1	TCAGATGCTCTCCCCTCCTC	CTACGGGCATACGGTTTGGT
FGFR2	AGCACCATACTGGACCAACAC	GGCAGCGAAACTTGACAGTG
FGFR3	TGCGTCGTGGAGAACAAGTTT	GCACGGTAACGTAGGGTGTG
FGFR4	GAGGGGCCGCCTAGAGATT	CAGGACGATCATGGAGCCT

SUPPLEMENTARY METHODS

Cell proliferation assay

Cells were seeded at an appropriate density of 1000-2000 cells/well in 96-well plates. Following a gradient dose treatment with the compounds for 5 days, cell proliferation was assessed using the sulforhodamine B (SRB, S1402-25G, Sigma-Aldrich, Darmstadt, Germany) assay for adherent cells or the methyl thiazolyl tetrazolium (MTT, M59609-5G, ABCONE, Shanghai, China) assay for suspended cells. The IC_{50} values were determined using GraphPad Prism software. To assess the synergistic effect of two compounds, the combination index (CI) was calculated using the CalcuSyn software, where a CI < 1 indicates a synergistic effect, CI = 1 indicates an additive effect, and CI > 1 indicates an antagonistic effect. Furthermore, the SynergyFinder platform (**https://www.synergyfinderplus.org/**) was employed to assess the synergistic effects of two compounds online by selecting HSA models. Specifically, synergy scores exceeding 10 indicate a synergistic effect, those below -10 denote an antagonistic effect, and scores within the range of -10 to 10 represent an additive effect.

3D cell culture assay

PC9/OR3 cells were transfected with a luciferase-expressing plasmid (pLenti-Luciferase-IRES-Neo) and seeded at 500 cells per well in a special U-shaped 96-well plate with low adsorption (7007, Corning, New York, USA). After 8 days of drug treatment, the cells were imaged by high-content imager (PerkinElmer, Waltham, USA) and the fluorescence was quantitatively analyzed by luciferase assay system (E1483, Promegra, Madison, USA).

Clonogenic assay

Cells were seeded at a density of 2000 cells/well in 6-well plates and treated for 7 days. After being washed with PBS, the colonies were fixed with 4% paraformaldehyde (P0099, Beyotime, Shanghai, China) at 4 °C for 15 min and then stained with 0.1% crystal violet (abs817172, Absin, Shanghai, China) in methanol for 15 min. Subsequently, colonies were washed and dried at room temperature, and imaged using an Axio Zoom V16 microscope (Carl Zeiss, Oberkochen, Germany). The colony formation rate was quantified by dissolving the crystal violet in 33.3% (v/v) acetic acid and measuring the absorbance at a wavelength of 600 nm with a microplate reader (TECAN, Männedorf, Switzerland).

Western blot assay

After treatment, cells were collected and lysed. Proteins were loaded onto SDS-PAGE gels and transferred onto polyvinylidene fluoride (PVDF, IPVH00010, Merck/Millipore, Darmstadt, Germany) membranes. The bands were probed with corresponding primary antibodies (**Supplementary Table S3**) at 4°C overnight and then incubated with corresponding secondary antibodies (**Supplementary Table S3**) at room temperature for 1 h. Images were captured by Tanon4600 using ECL chemiluminescence solution (34580, Thermo Fisher Scientific, Waltham, USA).

Transwell assay

Cell migration assays were conducted using 12-well plates with 8-μm pore transwell chambers. The lower chamber was filled with culture medium containing 10% FBS. PC9/OR3 cells were pretreated with the drugs in 6-well plates for 24 h, followed by digestion using trypsin. An equivalent number of cells was then resuspended in medium without FBS and added into the upper chamber. After 24 h, the cells that migrated to the bottom surface were stained with 0.1% crystal violet (abs817172, Absin). Color imaging was conducted using an Axio Zoom V16 microscope, and the quantity of migratory

cells was documented.

Wound healing assay

Cells were incubated overnight in 96-well plates, after which a linear scratch was created on the cell monolayer to induce wound formation. The cells were then treated with drugs and incubated for 24 h. Wound healing was monitored in real time using the Incucyte system (Sartorius, Göttingen, Germany), and the data were analyzed.

Small interfering RNA (siRNA) and plasmids transfection assay

Cells were seeded in 12-well plates at a density of 1×10^5 cells/well and incubated for 12 h. For transfection complex preparation, 4 μL RNAiMAX (13778150, Thermo Fisher Scientific) transfection reagent in 75 μL Opti-MEM (11058021, Thermo Fisher Scientific) was mixed with 50 nM siRNA in 75 μL Opti-MEM and subjected to room temperature incubation for 15 min. The transfection complexes were then added to cells and maintained for 48 h. siFGFR1 (sense: 5'-CCACACTGCGCTGGTTGAAAA-3'), siSTAT3 (sense: 5'-CACAAUCUACGAAGAAUCA-3'), and siPLK1 (sense: 5'-GACAGCCUGCAGUACAUAGdTdT-3') were designed and synthesized by Tsingke (Beijing, China).

The pCDNA3-PLK1(829) (#39845) plasmid was purchased from Addgene (Watertown, USA). The pCDNA3-STAT3 and pCDNA3-FGFR1 plasmids were constructed in our laboratory. The designated cells were transfected with the corresponding plasmids using the transfection reagent Lipofectamine™ 2000 (11668019, Thermo Fisher Scientific), according to the manufacturer's instructions.

RNA extraction and real-time quantitative PCR (RT-qPCR)

After treatment, cells were lysed using TRIzol (15596018, Invitrogen, Watertown, USA) and total

RNA was extracted. cDNA was synthesized using SuperScript III Reverse Transcriptase (RR036A, TaKaRa, Otsu, Japan). Hieff qPCR SYBR Green Master Mix (H6312080, YEASEN, Shanghai, China) was used to perform RT-qPCR. Specific primers were synthesized by Tsingke, with the sequence information presented in **Supplementary table S4**.

Immunofluorescence

NCI-H1975 cells were seeded on slides prepared in 6-well plates and cultured overnight for adhesion. Following siRNA transfection for 48 h, cells were fixed with 4% paraformaldehyde for 15 min at room temperature. Subsequent permeabilization was achieved using 0.1% Triton X-100, followed by blocking with 1% BSA for 1 h at room temperature. The cells were then incubated overnight at 4°C with anti-PLK1 (NB100-74502, Novus, Centennial, USA) and anti-P-STAT3 (ab76315, Abcam, Cambridge, UK) antibodies. The bound primary antibodies were detected by incubation with goat anti-rabbit Alexa Fluor 488- (1853312, Thermo Fisher Scientific) and goat anti-mouse Alexa Fluor 633-conjugated (1845042, Thermo Fisher Scientific) secondary antibodies for 1 h at room temperature. Finally, fluorescence images were acquired using a LSM710 confocal laser scanning microscope (Carl Zeiss).

Docking of protein-protein interaction

The PDB database was screened to acquire the 3D structure of PLK1 (PDB ID: 1Q4K), STAT3 (PDB ID: 6TLC) and JAK1 (PDB ID: 4EHZ). ClusPro v2.0 docking server (**https://cluspro.bu.edu**) was utilized to dock PLK1 with STAT3 and JAK1. ClusPro is a protein-protein rigid-body docking web tool which provides docked structures based on total energy functions of the complexes [1]. After docking analysis, PyMol was used for visual display.

Statistical analysis

All data are presented as the mean ± standard deviation (SD) or standard error of the mean (SEM). Without special explanation, statistical significance was evaluated using two-tailed unpaired student's t-test for comparisons between two independent groups and one-way ANOVA for univariate comparisons. $P < 0.05$ was considered statistically significant. All experiments were repeated at least three times. Spearman's coefficient was used to quantify the degree of correlation between two distinct variables.

SUPPLEMENTARY REFERENCES

1. Kozakov D, Hall DR, Xia B, Porter KA, Padhorny D, Yueh C*, et al.* The ClusPro web server for protein-protein docking. *Nature Protocols* 2017, **12**(2)**:** 255-278.
