# Supplementary material for: Targeting PLK1 potentiates the antitumor efficacy of EGFR-TKIs through inhibiting the JAK1/STAT3 pathway
Source: Cell Death Dis. 2026 Jan 15;17(1):41. doi: 10.1038/s41419-025-08220-9 (PMC12808168; doi:10.1038/s41419-025-08220-9)

Original Western Blots

Fig. 2E

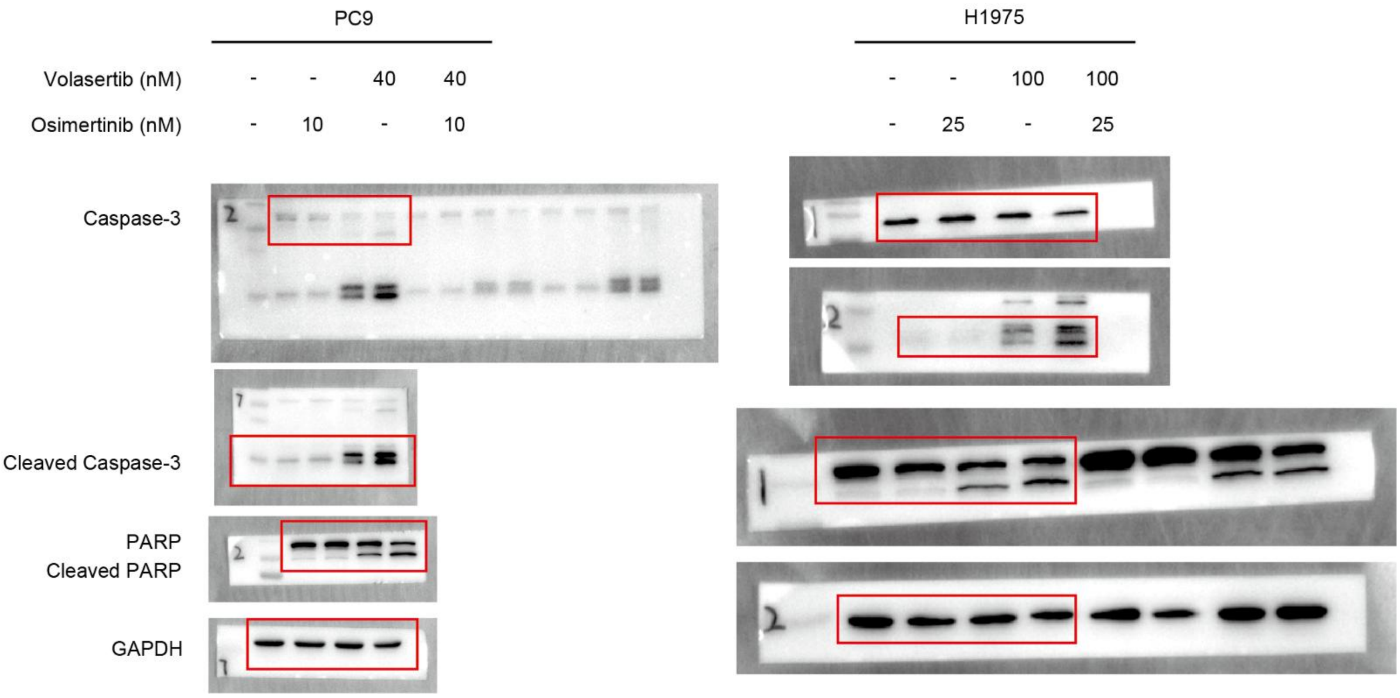

Fig. 3D

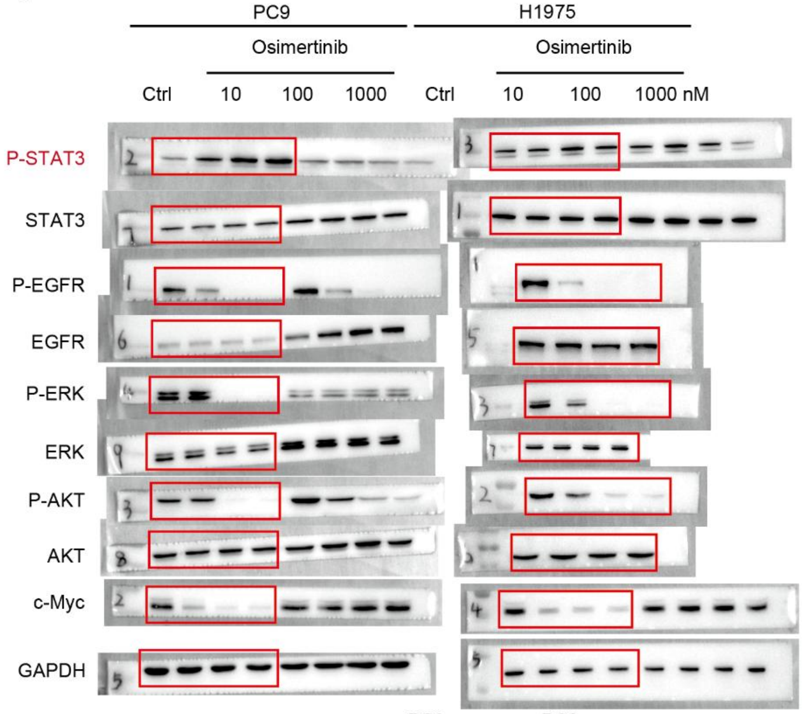

Fig. 3E

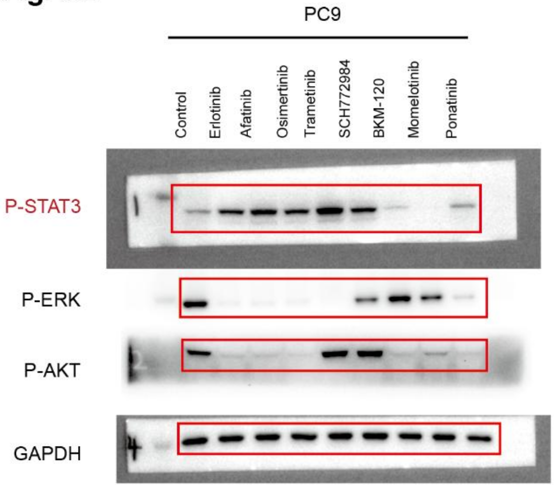

Fig. 3F

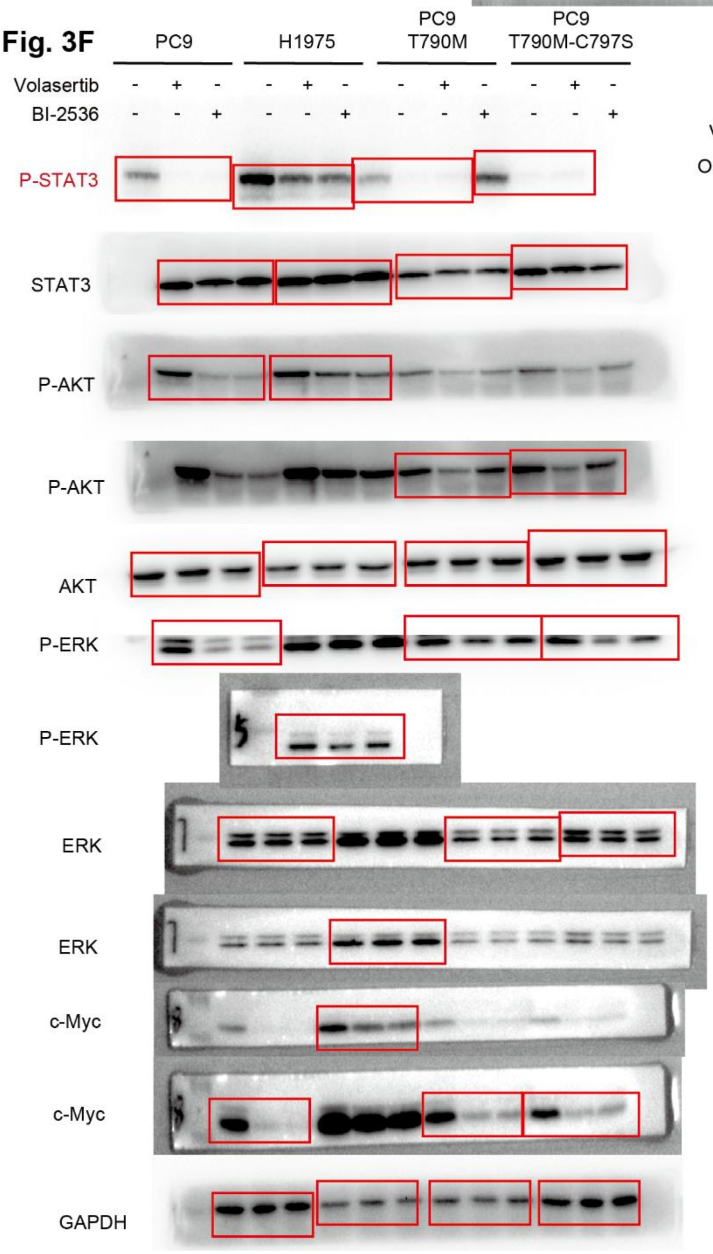

Fig. 3G

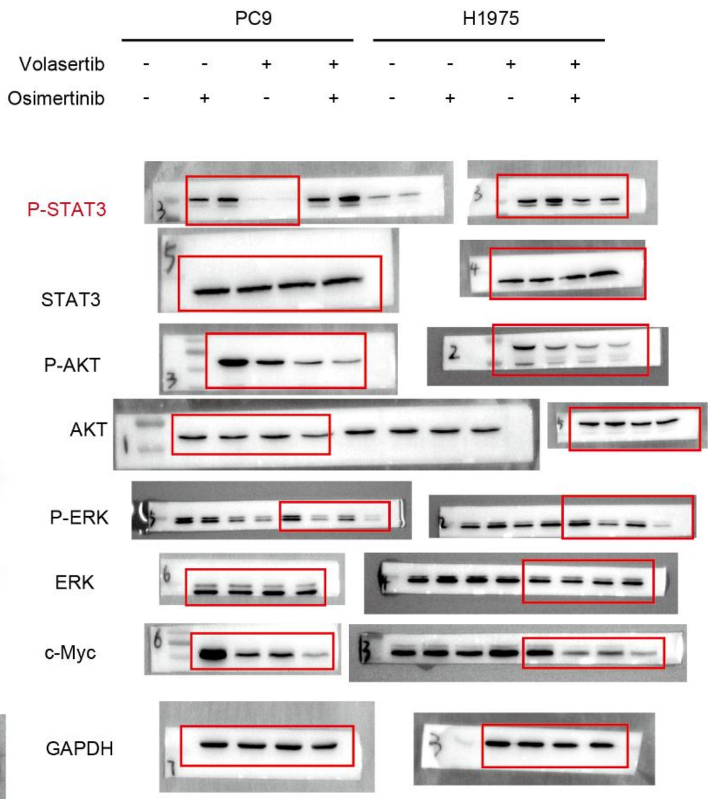

Fig. 3H

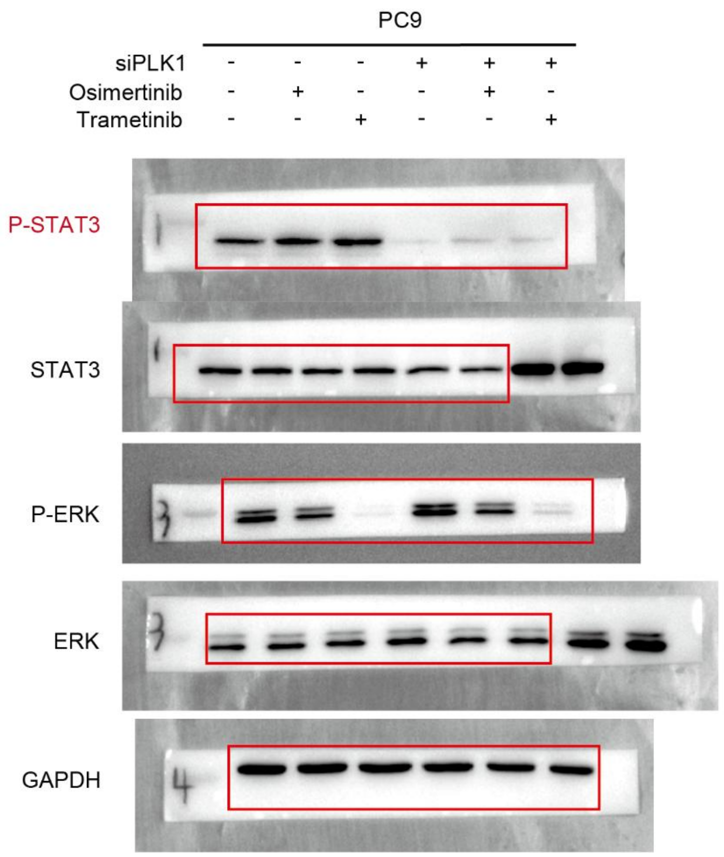
PC9
siPLK1 - - - + + +
Osimertinib - + - - + -
Trametinib - - + - - +
P-STAT3
STAT3
P-ERK
ERK
GAPDH

Fig. 3K

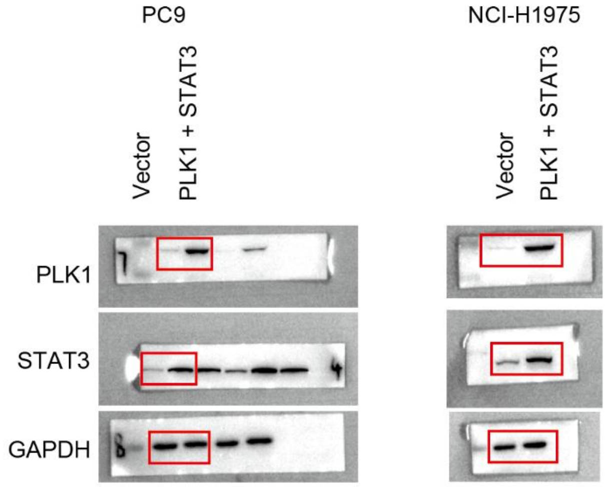
PC9
NCI-H1975
Vector
PLK1 + STAT3
Vector
PLK1 + STAT3
PLK1
STAT3
GAPDH

Fig. 4F

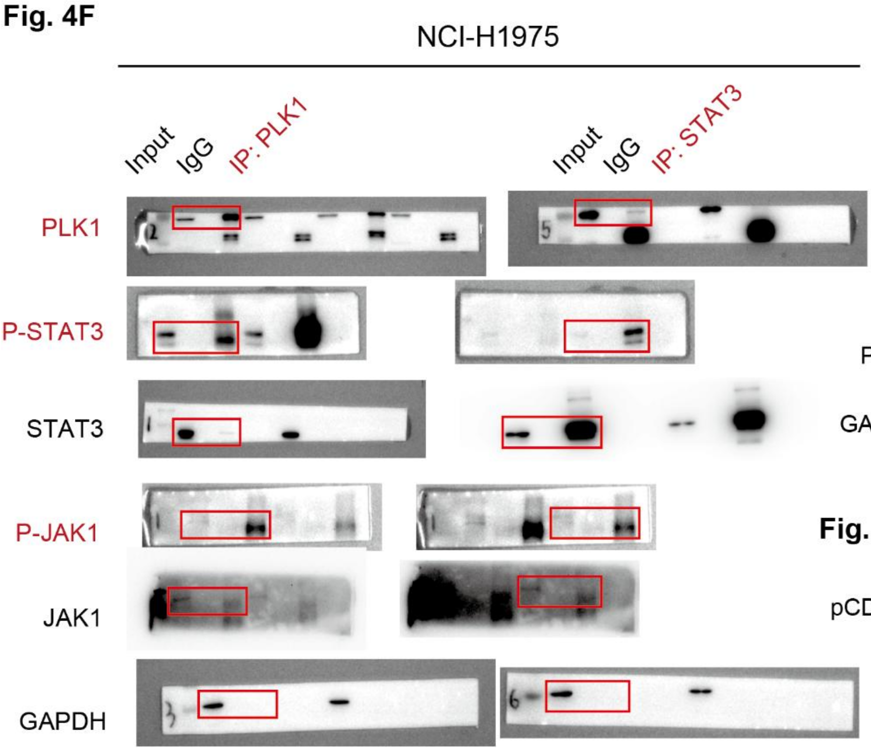

Fig. 4G

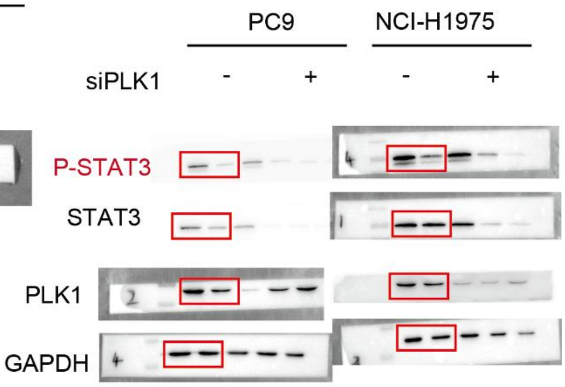

Fig. 4H

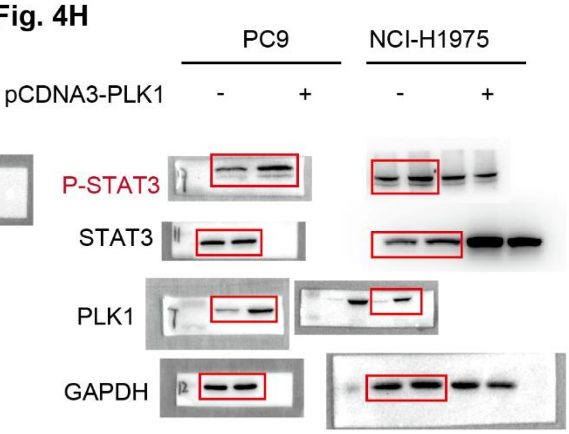

Fig. 4I

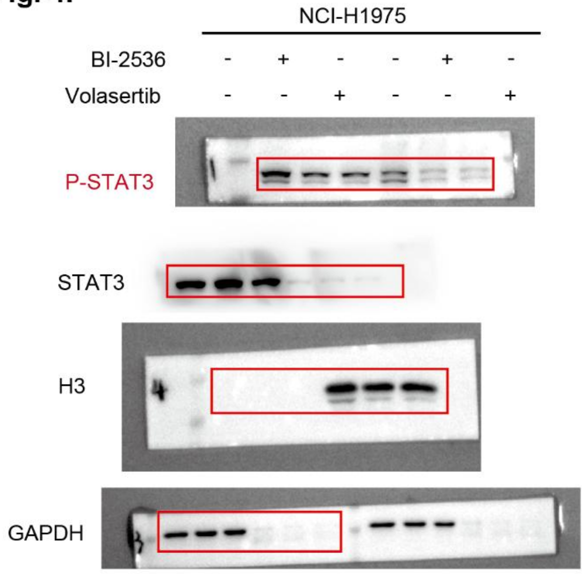

Fig. 4L

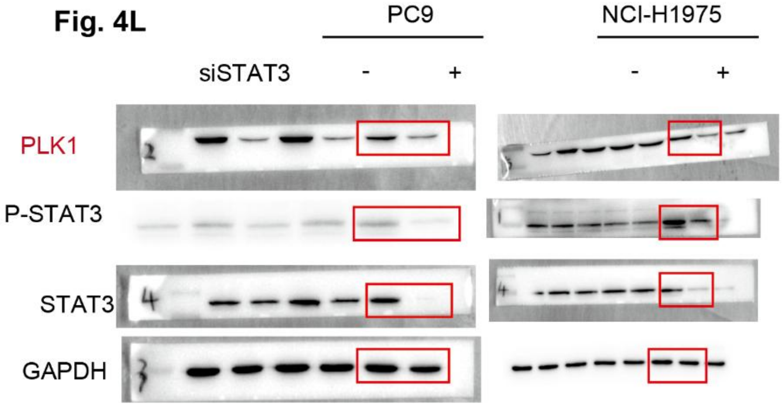

Fig. 5D

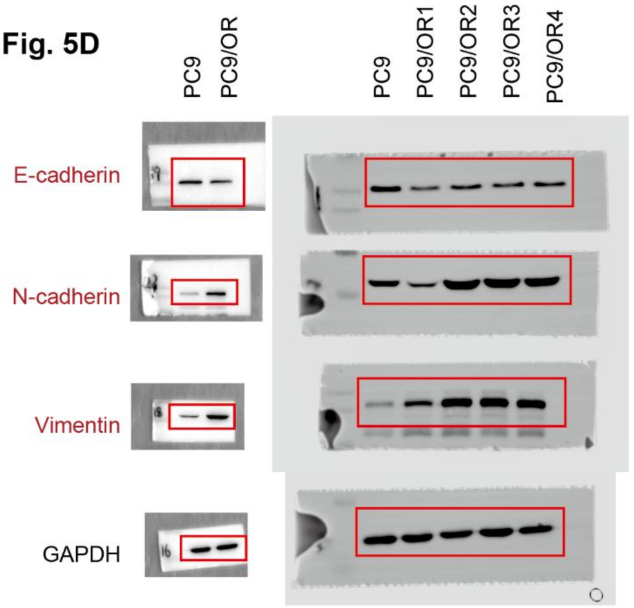

Fig. 5H

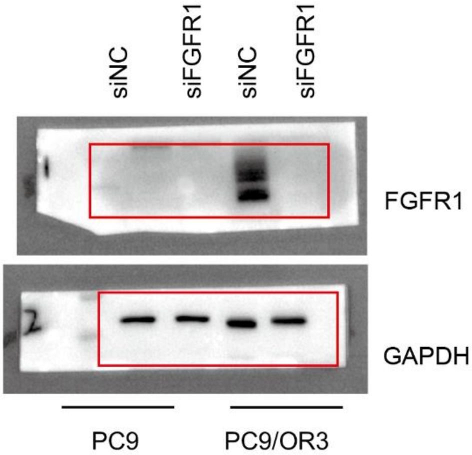

Fig. 5J

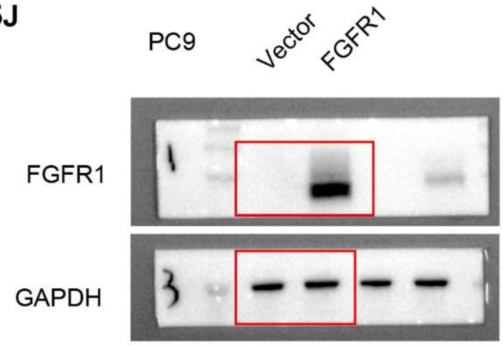

Fig. 5G

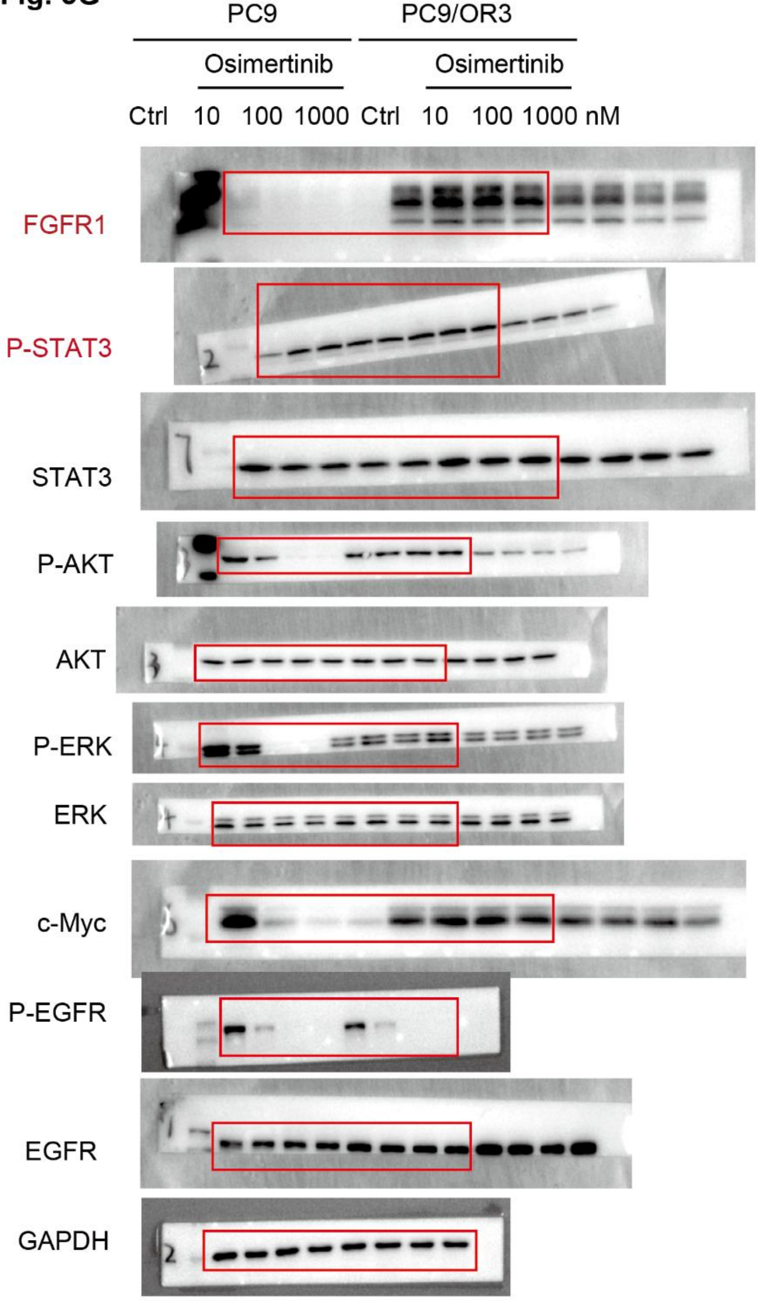

Fig. 5K

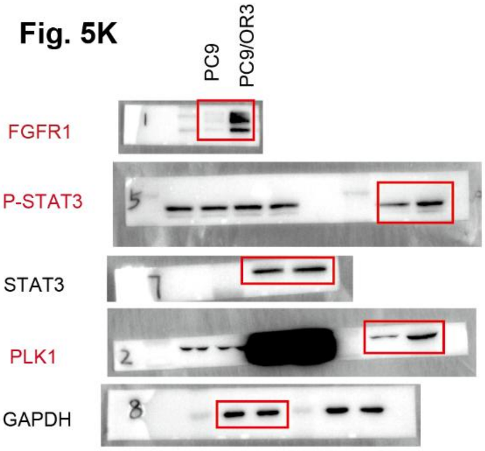

Fig. 5L

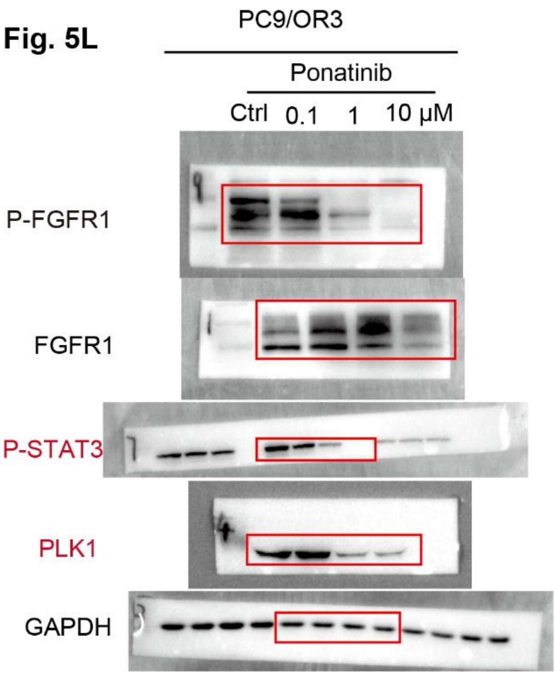

Fig. 5M

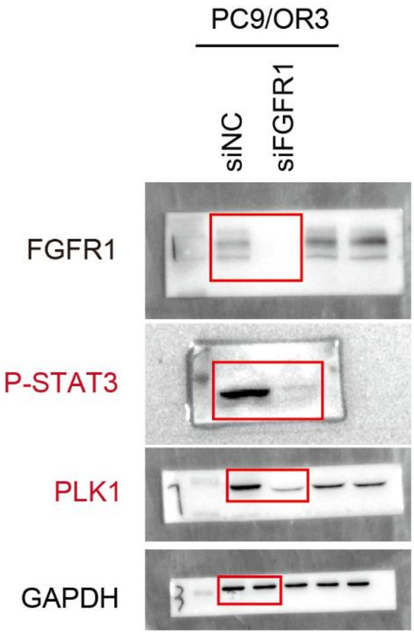

Fig. 5N

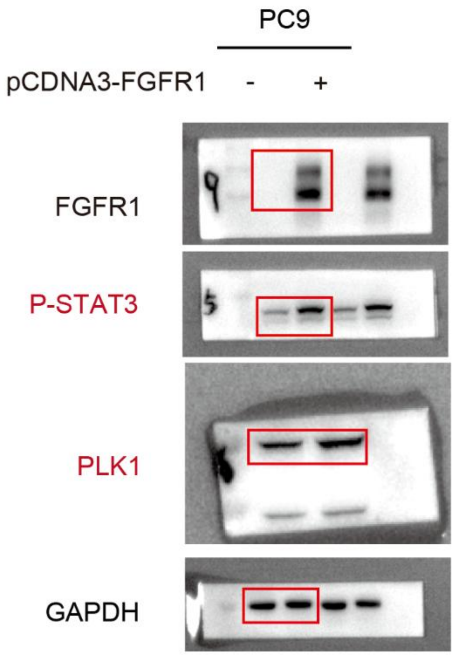

Fig. 5Q

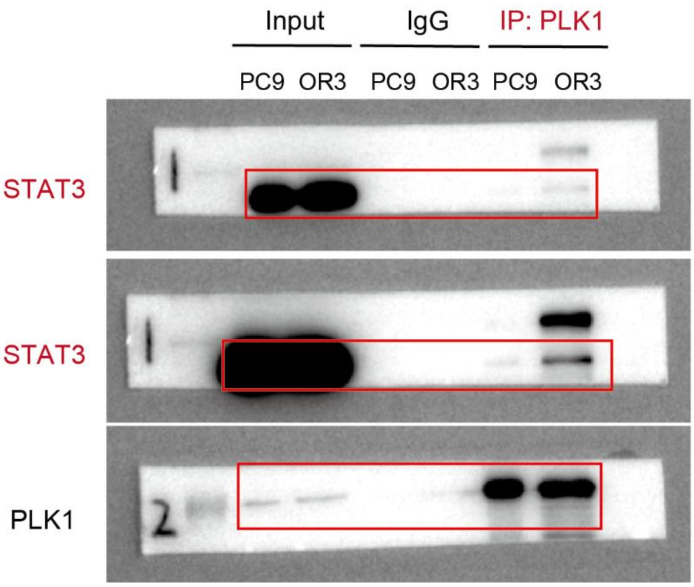

Fig. 5R

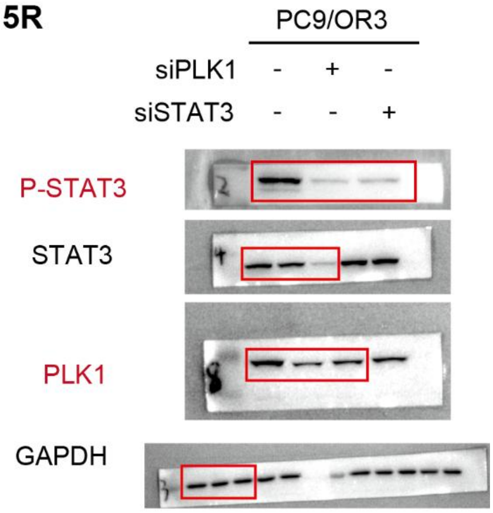

Fig. 6F

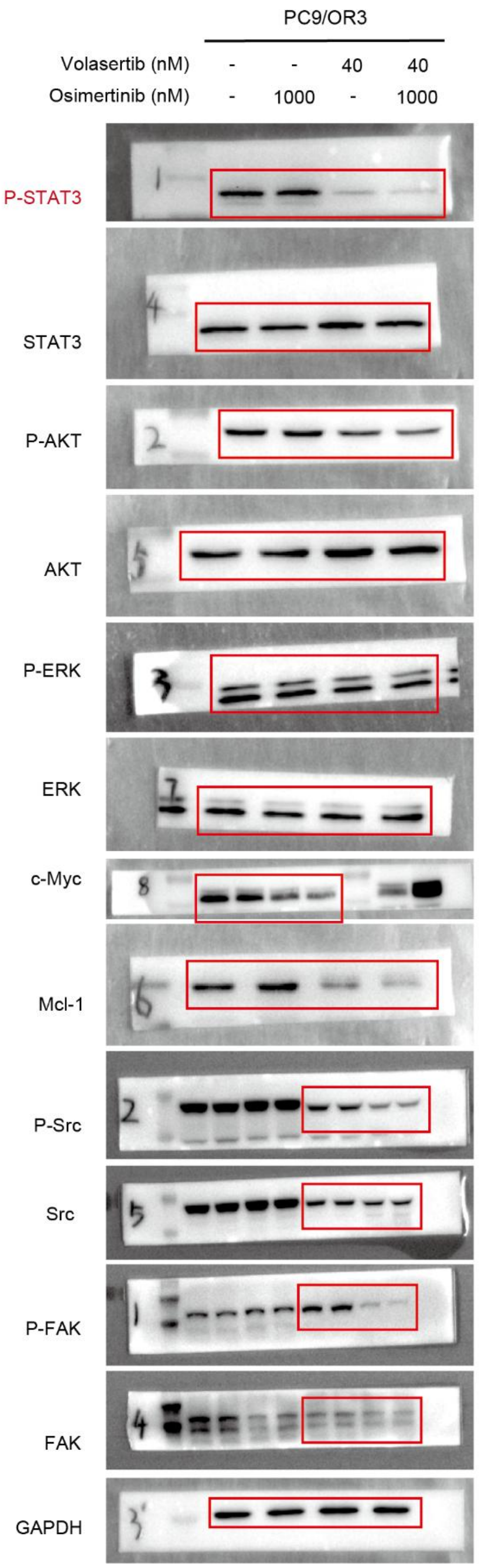
PC9/OR3
Volasertib (nM) - - 40 40
Osimertinib (nM) - 1000 - 1000
P-STAT3
STAT3
P-AKT
AKT
P-ERK
ERK
c-Myc
Mcl-1
P-Src
Src
P-FAK
FAK
GAPDH

Fig. 6J

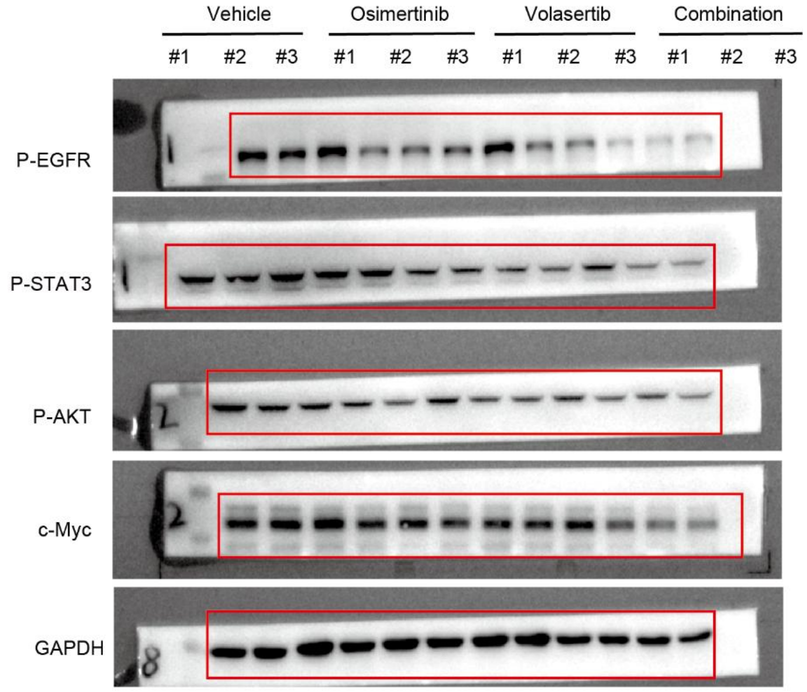
Vehicle
Osimertinib
Volasertib
Combination
#1 #2 #3 #1 #2 #3 #1 #2 #3 #1 #2 #3
P-EGFR
P-STAT3
P-AKT
c-Myc
GAPDH

Fig. S2A

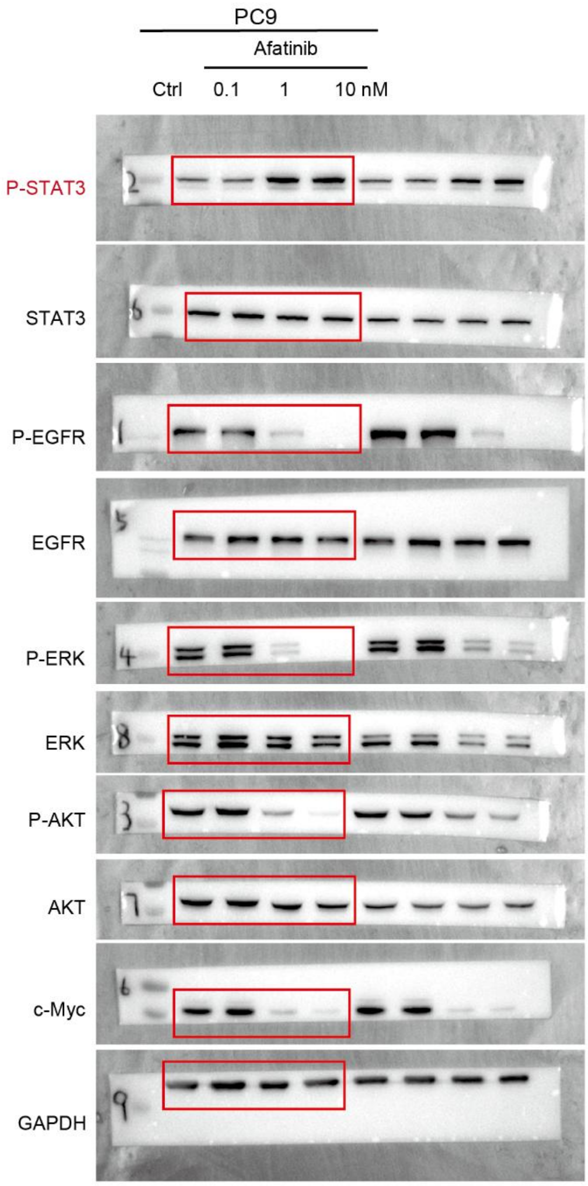

Fig. S2B

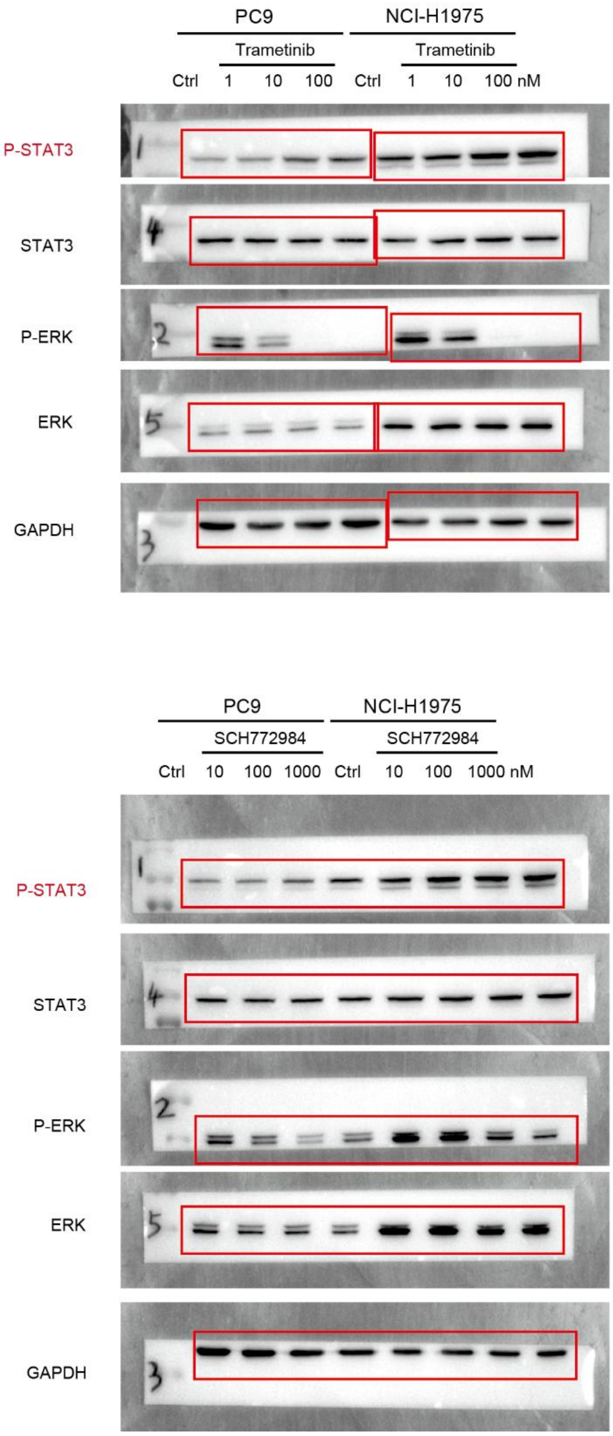

Supplement: Supplementary file 2 — Original Western Blots [file 41419_2025_8220_MOESM2_ESM.pdf]
